# Need for speed: evaluation of dilute and shoot-mass spectrometry for accelerated metabolic phenotyping in bioprocess development

**DOI:** 10.1007/s00216-021-03261-3

**Published:** 2021-03-31

**Authors:** Alexander Reiter, Laura Herbst, Wolfgang Wiechert, Marco Oldiges

**Affiliations:** 1 Institute of Bio- and Geosciences, IBG-1: Biotechnology, Forschungszentrum Jülich GmbH, 52425 Jülich, Germany; 2 Institute of Biotechnology, RWTH Aachen University, 52062 Aachen, Germany; 3 Computational Systems Biotechnology, RWTH Aachen University, 52062 Aachen, Germany

**Keywords:** LC-MS/MS, Matrix effects, FIA, Amino acids, *Corynebacterium glutamicum*, Metabolomics

## Abstract

With the utilization of small-scale and highly parallelized cultivation platforms embedded in laboratory robotics, microbial phenotyping and bioprocess development have been substantially accelerated, thus generating a bottleneck in bioanalytical bioprocess sample analytics. While microscale cultivation platforms allow the monitoring of typical process parameters, only limited information about product and by-product formation is provided without comprehensive analytics. The use of liquid chromatography mass spectrometry can provide such a comprehensive and quantitative insight, but is often limited by analysis runtime and throughput. In this study, we developed and evaluated six methods for amino acid quantification based on two strong cation exchanger columns and a dilute and shoot approach in hyphenation with either a triple-quadrupole or a quadrupole time-of-flight mass spectrometer. Isotope dilution mass spectrometry with ^13^C^15^N labeled amino acids was used to correct for matrix effects. The versatility of the methods for metabolite profiling studies of microbial cultivation supernatants is confirmed by a detailed method validation study. The methods using chromatography columns showed a linear range of approx. 4 orders of magnitude, sufficient response factors, and low quantification limits (7–443 nM) for single analytes. Overall, relative standard deviation was comparable for all analytes, with < 8% and < 11% for unbuffered and buffered media, respectively. The dilute and shoot methods with an analysis time of 1 min provided similar performance but showed a factor of up to 35 times higher throughput. The performance and applicability of the dilute and shoot method are demonstrated using a library of *Corynebacterium glutamicum* strains producing l-histidine, obtained from random mutagenesis, which were cultivated in a microscale cultivation platform.

**Supplementary Information:**

The online version contains supplementary material available at 10.1007/s00216-021-03261-3.

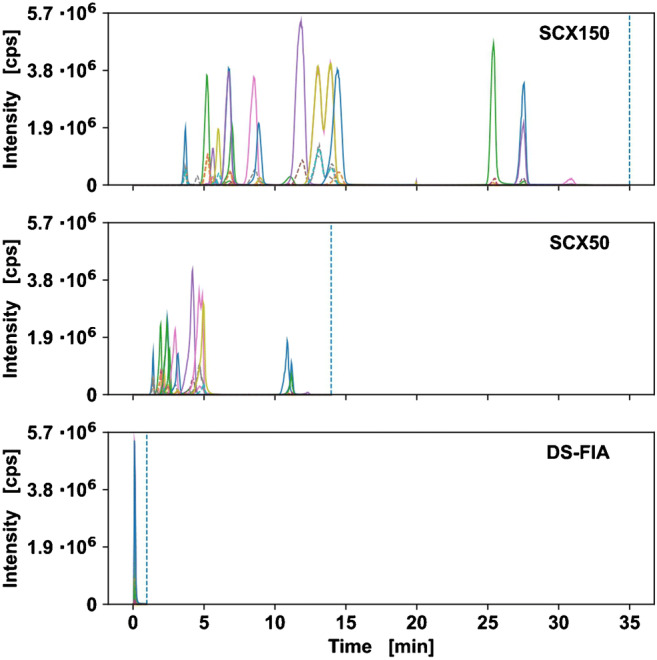

## Introduction

In order to pave the way for a sustainable economy and societal development, industrial biotechnology [1–3] is a major pillar in such transformation process [4–6]. Microbial production of valuable metabolites and other small molecule products plays an important role in order to supply chemical intermediates and precursors [7, 8], fine chemicals [9–11], and biopharmaceutically active target molecules [12–14]. A broad range of microbial production organisms are used comprising prokaryotes and eukaryotes, dependent of the chemical nature of the target molecule [15–17]. Despite the fact that a huge variety of microbial producers are in use, the largest part of industrial biotechnology processes is performed by a limited set of so-called platform organisms [18–24], such as *Escherichia coli*, *Corynebacterium glutamicum* (*C. glutamicum*), *Saccharomyces cerevisiae*, and *Bacillus subtilis* as well as fungi like *Aspergillus*, *Trichoderma*, and others.

Usually, the development of efficient microbial cell factories is characterized by generation of larger strain libraries, following either rational metabolic engineering approaches or non-targeted approaches by mutagenesis and screening [25–27]. Besides classical molecular biology techniques, modern workflows with genome editing and gene assembly tools are state of the art, which could be used to provide large libraries in short time [28–30]. This is followed by strain performance evaluation usually taking place in high-throughput micro plate cultivation setups, allowing to process hundreds or thousands of clones per week [31–33].

Meanwhile, microcultivation technology, such as minibioreactors [34] or microbioreactors [35], has been matured to mimic lab-scale bioreactor cultivations to a high extent [36, 37]. Such devices are often integrated in laboratory robotic systems to increase versatility and performance, providing process samples and process information in a comparable manner to traditional laboratory-scale bioreactors, but with elevated throughput in the order of up to 2 orders of magnitude. This is sufficient to handle typical size of strain libraries with approx. 500 to 1000 clones per week with end point determination by sacrifice sampling only. When using such technology supported by laboratory robotics for low volume process sampling, 48 parallel microcultivations can lead to approx. 400 samples in 2 days only [38].

For performance evaluation of the cultures, an adequate analytical method is required. Such method should ideally provide (a) selective detection of target compound and quantitative information, (b) robustness and accuracy in presence of sample matrix, (c) option for detection of by-product spectrum, and (d) sufficient throughput in order to cope with the accruing samples from screening stage [39]. For the sake of simplicity and throughput, the product is often measured by spectrophotometric assays, which are usually susceptible for automation and robotic assistance to provide the necessary throughput [40, 41]. Usually, such assays provide the result for the target molecule only, sometimes with limited quantitative quality of the data as well as no information about the by-product spectrum, which is of very high importance for further metabolic strain engineering [42].

To give an example, for high-throughput amino acid product screening, the ninhydrine assay is used quite frequently [43]. While very simple in application, the reaction is non-specific for an individual amino acid, i.e., it gives false positive results for any other amino acid. Moreover, the assay is difficult to perform quantitatively for an individual amino acid in the presence of more than one amino acid, which is indeed a typical challenge in the screening of amino acid producer libraries. In addition, the response factor is different for every amino acid, due to the sensitivity of the color developing reaction to the chemical nature of the respective amino acid. Other well-established analytical options for amino acid measurement, such as HPLC-FLD [44], CE-MS [45] or LC-MS/MS [46], are often not fast enough and need to be optimized in terms of speed and sample throughput, which can be enabled by microfluidic devices [47], chromatography optimization, or UPLC [48–50]. Despite the ease of photometric methods, mass spectrometric detection is expected to be superior with respect to individual resolution of co-eluting amino acids, method robustness to matrix, and quality of quantification especially when speeding up chromatographic separation.

In this contribution, we evaluated an established LC-MS/MS method for the detection and quantification of 19 proteinogenic amino acids with respect to the potential to significantly decrease sample run time by optimization of chromatographic conditions. Moreover, we also tested omission of chromatographic separation in a flow injection analysis (FIA) style approach with a dilute and shoot (DS) method. The two chromatographic methods, as well as the DS-FIA method, were tested in hyphenation mode with two types of tandem mass spectrometers, i.e., triple-stage-quadrupole (QqQ) and quadrupole time-of-flight (QqToF). It turned out that valid quantification was possible with the two chromatographic methods as well as the DS-FIA, when isotope dilution mass spectrometry (IDMS) was used for quantification. The developed DS-FIA-QqQ method was fully able to demonstrate the ability to screen even larger strain libraries with a speed of one sample/minute, contributing to debottleneck strain engineering and bioprocess development of microbial strain and production process development.

## Materials and methods

### Chemicals

The unlabeled amino acids l-aspartic acid (Asp), l-glutamic acid (Glu), l-serin (Ser), l-asparagine (Asn), l-threonine (Thr), l-glutamin (Gln), l-tyrosine (Tyr), l-glycine (Gly), l-proline (Pro), l-alanine (Ala), l-methionine (Met), l-valine (Val), l-phenylalanine (Phe), l-isoleucine (Ile), l-leucine (Leu), l-tryptophane (Trp), l-histidine (His), l-lysine (Lys), and l-arginine (Arg) were purchased from Sigma-Aldrich (Schnelldorf, Germany). The cell-free extract of ^13^C^15^N labeled amino acids was purchased from Sigma-Aldrich (Schnelldorf, Germany). UPLC/MS-grade methanol (MeOH) was obtained from Biosolve BV (Valkenswaard, Netherlands). LC-MS-grade ammonium acetate was purchased from Merck (Darmstadt, Germany). Acetic acid (Ph. Eur.) was obtained from Roth (Karlsruhe, Germany). Cultivation media components were purchased from Sigma-Aldrich (Schnelldorf, Germany) or from Roth (Karlsruhe, Germany). LC-MS-grade water was obtained from a Milli-Q water purification system (Merck Millipore, Burlington, MA, USA).

### LC-MS/MS

All standards were prepared in 50% MeOH (v/v) and stored at −80 °C. The ^13^C^15^N labeled cell-free amino acid mixture was diluted 1:4∙10^3^ with 50% MeOH (v/v) to a final concentration of 1.25–16.25 μM. Calibration standards were prepared as 100 μM stock solution. For the dilution series, the analytical stock solution was diluted with 50% MeOH (v/v) in sequential dilution series with 12 concentrations each. For the strong cation exchange methods (SCX), a logarithmic dilution series of 50, 25, 10, 5, 2.5, 1, 0.5, 0.25, 0.1, 0.05, 0.025, and 0.01 μM was used. For the DS-FIA methods, a linear dilution series of 16, 14, 12, 10, 8, 6, 4, 2, and 1 μM and subsequent logarithmic dilution series of 1, 0.5, 0.25, and 0.01 μM was applied. For method optimization, standards and samples were diluted with either 50% MeOH (v/v) or the corresponding mobile phase. For spiking experiments, medium was diluted accordingly prior to spiking with standard stock solution. For IDMS, standards and samples were diluted 1:2 with a 1:4∙10^3^ diluted ^13^C^15^N labeled cell-free amino acid mixture.

The analysis of amino acids was carried out with both an Agilent 1100 system with an Agilent 1260 autosampler and an Agilent 1260 Infinity system (Agilent Technologies, Waldbronn, Germany). For the column-based methods, SCX150 (150 × 2 mm, 5 μm) and SCX50 (50 × 2 mm, 5 μm) with a SCX guard column (4 × 2 mm, 5 μm) were used (Phenomenex, Torrance, CA, USA). Elution was carried out with 5% acetic acid (v/v) (solvent A) and 15 mM ammonium acetate (pH 6.0, adjusted with 100% acetic acid) (solvent B) at a flowrate of 400 μL min^−1^ and 60 °C. The elution gradient for the SCX150 methods was as follows: 0 min, 15% B; 10 min, 15% B; 16 min, 100% B; 28 min, 100% B; 30 min, 15% B; 35 min, 15% B. The elution gradient for the SCX50 method was as follows: 0 min, 15% B; 4 min, 15% B; 6 min, 100% B; 11.5 min, 100% B; 12 min, 15% B; 14 min, 15% B. For the DS-FIA methods, polyetheretherketone (PEEK) capillary was directly connected to MS with mobile phase consisting of 5% acetic acid (v/v) and 5% MeOH (v/v) at a flowrate of 400 μL min^−1^ and 21 °C. For the method optimization, the organic fraction (v/v) of the mobile phase was varied with 0%, 5%, 10%, and 15% (v/v). Injection volume for all methods was 5 μL.

Mass spectrometry was carried out with both a QqQ and QqToF (API4000, TripleTOF6600, AB Sciex, Darmstadt, Germany), both equipped with the corresponding TurboV ion source. Operated in positive ionization mode, the ion source voltage was set at 5.5 kV, the source temperature at 650 °C, curtain gas at 25 psi, and the support gases GS1/GS2 at 30 psi/70 psi. All gases, including collision gas set at 5 psi, were nitrogen. Compound optimization was performed with QqQ by direct injection of single unlabeled amino acids (see Supplementary Information (ESM_1)). Average product ion (PI) spectra were acquired by a collision energy ramp with a QqToF by direct infusion of unlabeled single standards dissolved in 50% methanol (v/v) (see ESM_2). The dwell time for all methods was 50 ms per mass transition for 38 (SCX150), 36 (SCX50), and 34 (DS-FIA) mass transitions.

Instrument control and data acquisition was performed with Analyst 1.6.3 for the QqQ and Analyst 1.7 TF for the QqToF (ABSciex, Darmstadt, Germany). The extracted ion chromatograms (XIC) of MRM and PI modes were automatically processed with the MQ4 algorithm of MultiQuant 3.0.3 (ABSciex, Darmstadt, Germany). Data processing was conducted with Python 3.7 and the packages pathlib 1.0.1, pandas 1.0.2, numpy 1.18.1, matplotlib 3.1.1, seaborn 0.10.0, scipy 1.4.1, and statsmodels 0.11.0.

### Validation

Calibration was performed by least squares approximation for non-weighted linear regression of the ^12^C^14^N/^13^C^15^N peak area ratio with the corresponding concentration. Appropriate concentration ranges were selected based on expected concentrations of highly diluted cultivation supernatants. The determination of range and linearity was conducted by shortening the calibration based on precision for lower boundary (RSD < 20%) and coefficient of determination *r*^2^ for the upper boundary (*r*^2^ > 0.99). The response factor (*m*) is the slope of the linear regression *y*(*x*) = *m* ∙ *x* + b. Tests for statistical significance based on a *t*-distribution with a degree of freedom df = *k* − 2 and a probability of error *α* set to *α* = 0.01 were conducted for the intercept (H_0_: *b* = 0). To evaluate and compare the performance of the methods, the process standard deviation coefficient *V*_C_ is used [51]:

1 $$ {V}_{\mathrm{C}}=\frac{1}{m\cdot \overline{x}}\cdot \sqrt{\frac{1}{k-2}{\sum}_{i=1}^k{\left({y}_i-{\hat{y}}_i\right)}^2}\cdot 100\% $$

Here *m* is the response factor and $\overline{x}$ the mean of the regression concentration range. *k* represents the number of concentration levels, *y*_*i*_ the measured response, and ${\hat{y}}_i$ the estimated value by the regression model. The method detection limit (MDL) is also used to evaluate the performance of the methods and described as follows [52]:

2 $$ \mathrm{MDL}={t}_{\alpha}\cdotp {c}_{LB}\cdotp \frac{s_{LB}}{y_{LB}} $$

The *t*-value *t*_α_ is a function of df = *n* − 1 and *α* = 0.01. *c*_*LB*_ denotes the concentration, *s*_*LB*_ the standard deviation, and *y*_*LB*_ the response (area ratio) of the linear range lower boundary (*LB*). The practical quantitation limit (PQL) was set to PQL = 5∙MDL. Evaluation of method robustness, accuracy, and precision was prepared by spiking 6 μM amino acid stock solution in H_2_O, CGXII, and MOPS-buffered CGXII prior to IDMS dilution. Recovered pools by all methods in different media were statistically compared by mean (H_0_: *c*_1_ = *c*_2_ = *c*_3_) in two approaches: (a) pools of one media by all methods for accuracy and (b) pools of all media by one method for robustness. One-way ANOVA and the post hoc Holm-Bonferroni test with *α* = 0.05 and df = *n* − 1 were used to consider errors by multiple testing. For direct method comparison of precision, recovered pools were evaluated by RSD with a threshold of RSD < 20%. All validation experiments were performed in four technical replicates.

### Strain library, cultivation media, and cryopreservation

All cultivations were performed with defined CGXII media [53] containing per liter of distilled water: 20 g d-glucose, 20 g (NH_4_)_2_SO_4_, 5 g urea, 1 g KH_2_PO_4_, 1 g K_2_HPO_4_, 13.25 mg CaCl_2_∙2 H_2_O, 0.25 g MgSO_4_∙7 H_2_O, 0.2 mg biotin, 30 mg protocatechuic acid, 10 mg FeSO_4_∙7 H_2_O, 10 mg MnSO_4_∙H_2_O, 1 mg ZnSO_4_∙7 H_2_O, 0.313 mg CuSO_4_∙5 H_2_O, and 0.02 mg NiCl_2_∙6 H_2_O. The medium was buffered with 42 g L^−1^ 3-(N-morpholino)-propanesulfonic acid (MOPS) and pH was adjusted to 7.0 with 4 M NaOH.

SenseUp GmbH (Jülich, Germany) provided a master cell bank (MCB) of 96 *C. glutamicum* His producer strains obtained from random mutagenesis which was stored at −80 °C. The working cell bank was prepared in microtiter plates. For this purpose, 96 square deepwell plate cultures in buffered CGXII medium were inoculated with 20 μL of the MCB. Cultivation took place with a filling volume of 500 μL at 30 °C, 300 rpm, and 80% humidity in an ISF1-X Shaker (Kuhner, Birsfelden, Switzerland) with a 50-mm shaking diameter. The cell suspension was harvested during exponential growth, distributed to sterile microtiter plates, and subsequently diluted 1:2 with 500 g L^−1^ sterile glycerol solution. The single-use microtiter plate aliquots were sealed with aluminum foil and stored at −80 °C.

### Cultivation, sampling and sample processing

Microscale batch cultivations of *C. glutamicum* strains were performed at 30 °C, 1300 rpm, and 80% humidity in a BioLector system (m2p-labs GmbH, Baesweiler, Germany). The cultivation system is integrated into a liquid handling robot (Tecan Group, Maennedorf, Switzerland), which was used for inoculation, sampling, and sample processing. A 48-well flowerplate with optodes for pH and dissolved oxygen measurement (MTP-48-BOH 1, m2p-labs GmbH, Baesweiler, Germany) was filled with 780 μL buffered CGXII medium per well, inoculated with 20 μL from the respective well of the cryopreserved stock and manually sealed with sealing foil for automation (F-GPRS48-10, m2p-labs GmbH, Baesweiler, Germany). For replicate cultivations, an integrated pre-culture strategy was established. Pre-culture and main culture conditions were identical to microscale batch cultivations described above. The pre-culture for the corresponding strain was cultivated in the first row of the flowerplate. After reaching a predefined backscatter, the main culture wells were filled with 4 °C cooled CGXII medium and column-wise inoculated with the corresponding pre-culture.

For sampling, a total volume of 950 μL was aspirated from individual cultivation wells, deposited in a 2 mL deepwell plate and centrifuged for 5 min with 3220 g at 4 °C. 200 μL of the supernatants was transferred to a microtiter plate on a cooled carrier set to 4 °C and manually sealed with self-adhesive aluminum foil after all cultivations were finished. For LC-MS preparation, the samples were diluted using 50% MeOH (v/v) in three consecutive steps: 1:10, 1:10^2^, and 1:10^3^. For IDMS-DS-FIA-MS/MS analysis, 50 μL of the 1:10^3^ dilution was mixed with 50 μL of internal standard. The samples were stored at −20 °C outside the liquid handling platform until analysis.

## Results and discussion

The aim of this study was the development of an accelerated mass spectrometric method to accurately and precisely measure amino acids in microbial cultivation supernatants. Current development of accelerated LC-MS methods results in ever-shorter analysis times and so-called FIA-MS methods without a separation column have been developed. With respect to bioprocess development, the DS approach was used in an untargeted study to classify yeast mutants for functional genomics by using their metabolic footprint [54]. In addition, a FIA-MS method was applied to profile intracellular metabolites with a QqToF mass analyzer to increase the sample throughput for 96-well micro plate cultivations [55]. Subsequently, the method was further developed into a real-time metabolome profiling application [56]. However, absolute quantification in a targeted approach still presents a major bottleneck for DS-FIA applications due to missing sample clean up and presence of ion suppression.

Here, we present a DS-FIA method to accurately and precisely quantify amino acids in supernatants even in strongly buffered cultivation media, which are typically used in microscale cultivation. For this purpose, we optimized a classical LC-MS/MS method with respect to run time and subsequently to handle ion suppression issues, deriving from the use of highly concentrated buffer molecules. The presented methods were validated by analytical key performance indicators. The application of the DS-FIA approach is demonstrated by screening a His-producing *C. glutamicum* library of 96 mutants to find the most promising producer by volumetric productivity (*P*_V_). The results of the method development and case study show a focus on proteinogenic amino acids, but the approach could be generalized to compounds with amino acid functionalization in general.

### Method optimization

The developed methods for ultra-fast quantification of amino acids were started based on the method for amino acid determination by Thiele et al. [46], which is a targeted approach for proteinogenic amino acids. The quantification of Cys was excluded due to the suspected oxidation to l-cystine.

**Fig. 1:**
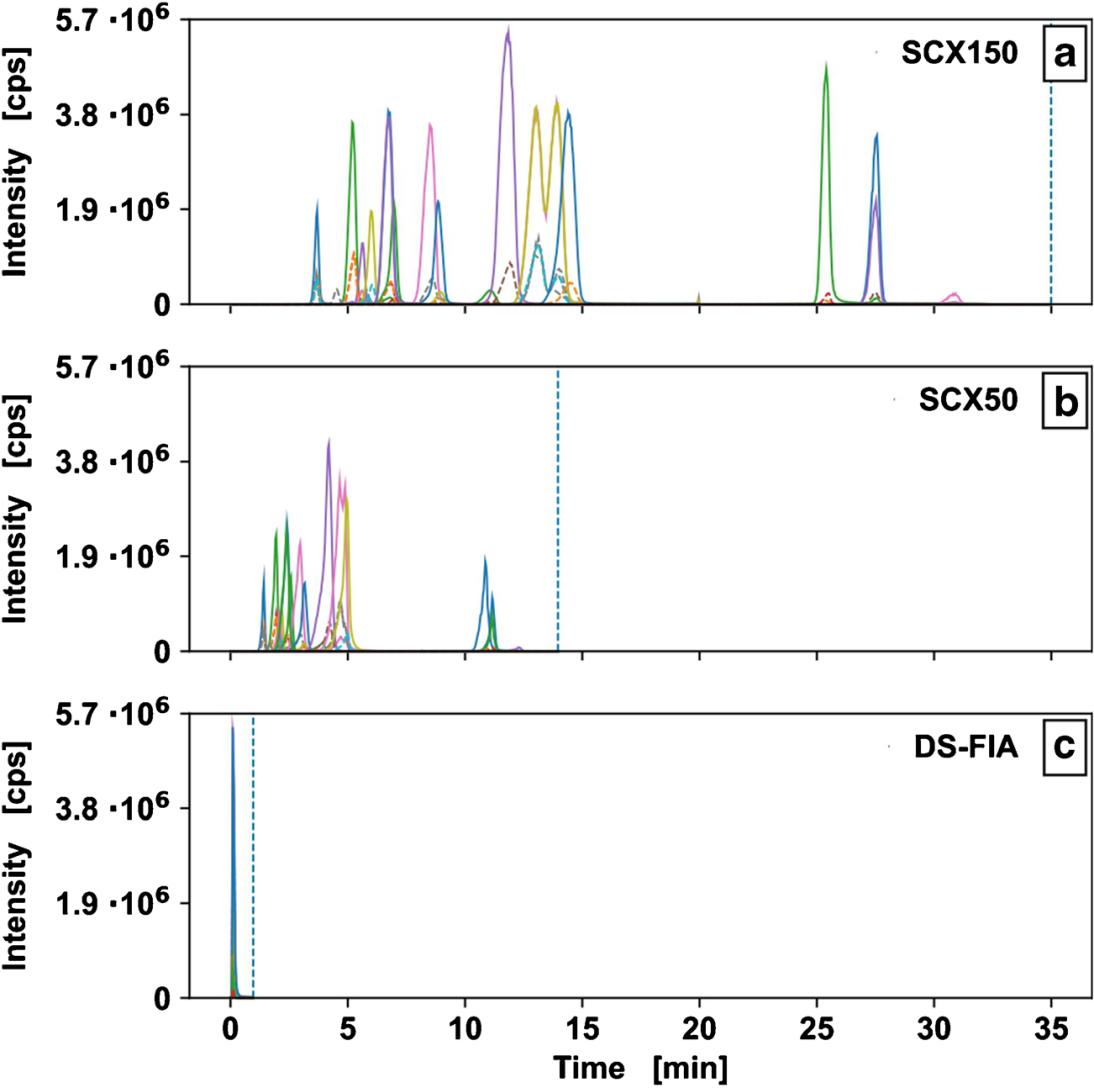
Extracted ion chromatograms of three methods for the quantitation of 19 proteinogenic amino acids with IDMS. Two LC-MS/MS methods with SCX150 mm (**a**) or SCX50 mm (**b**) columns and an analysis time of 35 min/sample respectively 14 min/sample. DS-FIA method (**c**) for ultra-fast quantitation with an analysis time of 1 min/sample; 5 μL injection volume of 50 μM amino acid standard solution diluted 1:2 with ^13^C^15^N labeled amino acids

In a first round of optimization, the analysis time of the original method was reduced from 75 to 35 min. This was possible by basic chromatographic method optimization, reducing the ammonium acetate concentration from 75 to 15 mM, increasing the flow from 0.2 to 0.4 mL min^−1^ and the column temperature from 40 to 60 °C as well as proper adjustment of elution gradient leading to the novel SCX150 method (Fig. 1a). Subsequently this method could be further accelerated down to 14 min by shortening the column length to 50 mm (SCX50) and adjusted elution gradient (Fig. 1b). LC parameters in form of retention time, peak width, tailing factors, and relational parameter like resolution for isobaric analytes can be found in S1 and S2 in ESM_3. Although peak width at 5% and 50% peak height did decrease, fronting was promoted for all analytes (see S1 in ESM_3). Independent of the method, analytes showed an RSD of retention times < 20% (see S1 in ESM_3).

Although the adjusted gradient of the SCX50 methods allowed for an analysis runtime of 14 min/sample, Ile and Leu were no longer separated, due to their similar chromatographic properties. Moreover, their identical collision-induced dissociation (CID) fragmentation pattern did not allow for a separation based on the mass-to-charge (m/z) ratio with resulting in at least one superimposed analyte signal (see Charts 10, 11 in ESM_2). The complete omission of any chromatographic column allowed for an analysis runtime of 1 min/sample (Fig. 1c) and further increased the possible sample throughput significantly. However, with the loss of chromatographic separation, Gln and Lys could not be determined independently, due to their isobaric nature. Although Gln and Lys have a different side chain, their CID fragments show the same m/z ratio (see Charts 6 and 12 in ESM_2).

When stable isotope labeling of compounds comes into play for application of IDMS for precise quantification, a further level of complexity is added especially for the DS-FIA methods. Hence, it is necessary to address the selection of the labeled isotopes and their MS/MS mass traces especially for DS-FIA applications. In general, ^13^C and/or ^15^N stable isotope labeled amino acids could be used for IDMS. With the prospect of automation and increased throughput due to accelerated sample processing and analysis, the use of standardized commercial and defined mixtures of amino acids with stable isotope labeling is preferred.

While there are algal-based ^13^C labeled amino acids’ mixtures, they are in general limited to 16 proteinogenic amino acids. On the other hand, mixtures of 20 amino acids are usually limited to ^15^N as well as ^13^C^15^N variants. For the sake of commercial availability of pure amino acid mixtures, ^13^C^15^N labeled amino acids were applied in this study. Nevertheless, the general application of ^13^C labeling is possible for the developed method. Sources of ^13^C labeled amino acids might origin from microbial cultivation with ^13^C-labeled d-glucose [57] or from labeled algal extracts [58].

The application of stable isotope labeled ^13^C^15^N or ^15^N labeled amino acids for IDMS comes along with an interference in fragmentation patterns of Asn and Asp as well as Gln and Glu. While the unlabeled analytes Asn and Asp can be distinguished by m/z in Q1, their ^13^C^15^N or ^15^N labeled equivalents cannot. Here, the m/z = 139 of ^13^C^15^N Asn/Asp interferes with ^13^C^15^N Ile/Leu resulting in a common peak for the internal standard of Asn, Asp, Leu, and Ile. Therefore, the common peak area of ^13^C^15^N Asn/Asp/Ile/Leu is used as normalization factor for both unlabeled single analytes Asn and Asp and the unlabeled peak area of Ile/Leu. The same challenge occurs for ^13^C^15^N Gln and ^13^C^15^N Glu, where the common peak area of ^13^C^15^N Gln and ^13^C^15^N Glu is used as normalization factor for Glu. A similar situation for Gln and Lys is solved by using the peak area of ^13^C^15^N Lys for the quantification of overlapping Gln and Lys.

Although no column was used in the DS-FIA approach, the DS-FIA signals showed little peak broadening and tailing (see S1 in ESM_3). Increasing capillary length or decreasing flow proportionally increased the broadening effect, showing that this is an effect of longitudinal diffusion, which can be used in an advantageous manner. For multi-compound MS/MS measurements, a specific dwell time per analyte is necessary, which requires a certain peak width to have sufficient data points for all target analytes. To achieve this, a 1 m PEEK capillary resulted in a slight, but sufficient peak broadening, much less than chromatographic broadening in chromatography columns. For 34 MS/MS transitions with dwell times of 50 ms, approx. 7–9 data points could be acquired per individual peak. In an ideal case, 15 data points/peak would be preferred, but 7–9 data points/peak provided sufficient peak integration quality for a rapid screening method. In total, 19 unlabeled and the respective 19 stable isotope labeled counterparts for IDMS were measured in a 1 min run using QqQ and QqToF.

Usually, the overall signal intensity is related to peak width, and for the same concentration, a narrower peak should result in higher absolute intensity. In contrast, in a multi-component analysis, the intensity of analytes might be reduced with smaller peak width, since co-elution of molecules might promote charge competition [59, 60], resulting in signal suppression based on molecule polarity and size [61]. Such signal suppression effect can be observed when decreasing the column length from SCX150 to SCX50 (Fig. 1a, b), showing very small change, from 5.7∙10^6^ to 3.8∙10^6^ for the shorter SCX50 column with chromatography. Consequently, a much higher peak intensity was expected for the DS-FIA sample (Fig. 1c), due to very little peak broadening. Strikingly, this is not observed and is most likely a consequence of the co-elution and signal suppression in the DS-FIA approach.

Compared to previously published method with 75 min run time [46], the analysis runtime is now reduced by factor of approx. 2, 5, and 75 for the methods SCX150, SCX50, and DS-FIA, respectively. The same authors already showed an acceleration of the original method for amino acid determination [62] with a runtime of 18 min, with constant buffer concentration of 75 mM, an upper linear range of 5 μM for 150 mm SCX column. The improved method in this contribution was able to reduce column length to 50 mm and preserve 25 μM as the upper linear range, while reducing ammonium acetate concentration down to 15 mM in the mobile phase. The latter is of high benefit in terms of salt loading for the ion source and potential issues of ion suppression in heavy salt-loaded cultivation supernatants.

At the start of the DS-FIA development, water was used for sample dilution. When applying the DS-FIA method with supernatant samples from cultivation with *C. glutamicum* diluted in H_2_O, a good recovery in the range of 90–100% was observed for most components, except for Gly, which showed a low recovery (up to 62% only) and high RSD (> 20%) (data not shown). It seems that the sample matrix contains compounds that interfere with the electrospray ionization (ESI), taking into account that no chromatographic separation is taking place. In the SCX150 and SCX50 methods, chromatography allows the separation of non-volatile salts and buffer substances from the analytes before the ESI process, which is not present in the DS-FIA approach. Especially the absence of organic content in the sample and mobile phase as well as its high salt content and the presence of MOPS buffer in concentrations up to 200 mM moved into the focus of further DS-FIA method optimization.

Hence, method optimization was targeted to reduce potential negative impact of ion suppression in the DS-FIA setup. The mobile phase composition can influence the ionization of all analytes and was chosen as an optimization target. The influence of mobile phase in ESI has been widely studied with respect to organic fraction, additives and pH [63, 64]. In this case, two questions arised: (a) can the ion suppression effects be reduced by increasing droplet volatility and reducing surface tension and (b) are the analyte ions properly ionized in the presence of buffer molecules. Gly, Ala, and His were chosen as model analytes with different molecular weights.

**Fig. 2:**
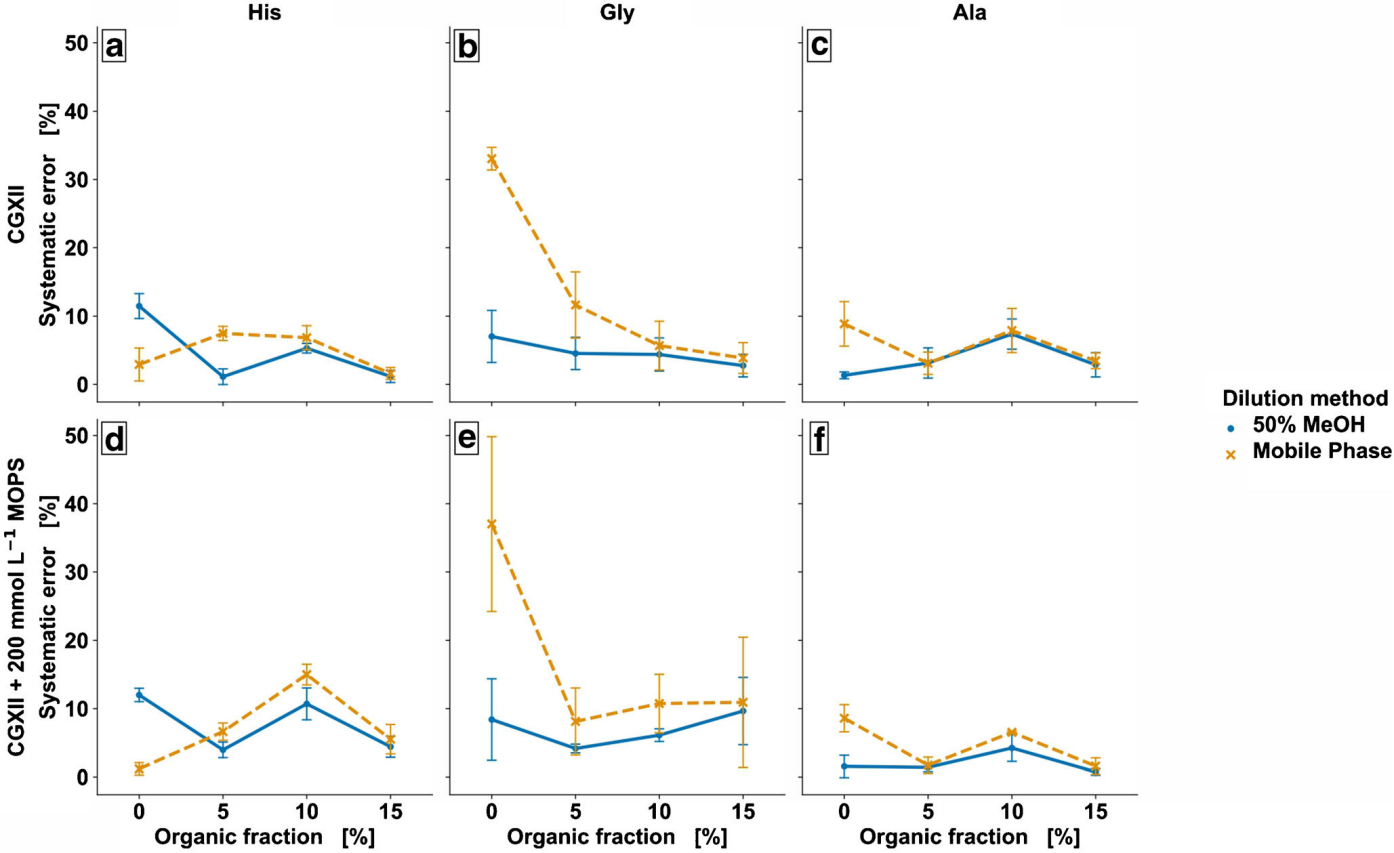
Error analysis by variation of organic fraction (v/v) in mobile phase for CGXII (**a**–**c**) and 200 mM MOPS-buffered CGXII (**d**–**f**) cultivation media demonstrated for His (**a**, **d**), Gly (**b**, **e**) and Ala (**c**, **f**). The systematic error represents the deviation from recovery in % based on the correspondingly diluted calibration series (*n*_analytical_ = 3)

To test the potential positive impact of organic fraction in calibration standards and media, they were either diluted with 50% MeOH (v/v) or the corresponding mobile phase. Unbuffered and buffered cultivation media were diluted by a factor of 1:10^3^ and spiked with 6 μM amino acid standard stock solution prior to 1:2 IDMS dilution. The systematic error represents the absolute deviation from recovery in % based on the corresponding calibration series (*n*_analytical_ = 3). Figure 2 shows the systematic error of quantification as a function of the MeOH content in the mobile phase for His, Gly, and Ala in CGXII standard medium (Fig. 2a–c) and CGXII plus 200 mM MOPS buffer (Fig. 2d–f) for dilution in 50% MeOH or mobile phase.

In general, the dilution with 50% MeOH showed smaller systematic deviation (Fig. 2a–f) compared to the dilution with mobile phase, but effect was small for Ala and His. Strikingly, there was large difference for Gly, showing error above 30% for the dilution in a fully aqueous phase only (Fig. 2b, e). It seems that robustness of ESI was reduced for Gly without any organic fraction when dilution was done with mobile phase only. However, a small portion of organic solvent methanol showed stabilizing effect and systematic error dropped rapidly down to approx. 10% and below for all three compounds.

The decrease in deviation for Gly with increasing organic fraction shows the benefit of organic modifier by decreasing surface tension and improving evaporation properties. This is supported by the data of the dilution with 50% MeOH (Fig. 2a–f, blue data), showing low deviation. As a result, dilution of samples with 50% MeOH was performed and an organic fraction of 5% MeOH in the mobile phase was chosen.

Besides the organic content of the mobile phase, potential negative effects originating from the presence of strong buffer, such as MOPS seem to be particularly evident for Gly (Fig. 2b, e). As a non-volatile substance with a molecular weight of *M*_MOPS_ = 209 g mol^−1^ it shows the typical properties of an ion suppressing component in the sample matrix.

**Fig. 3:**
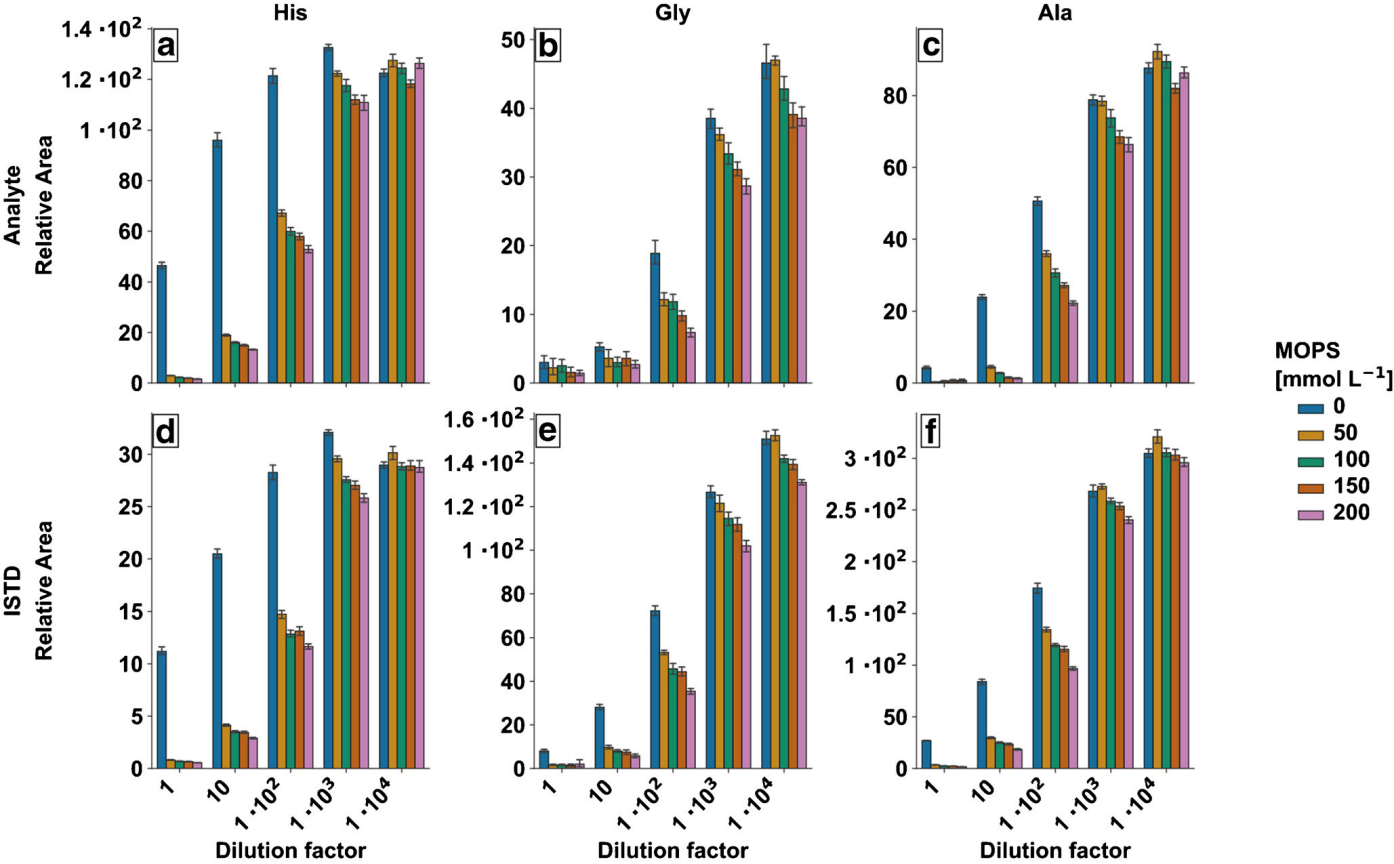
Analysis of matrix effects demonstrated by relative area of unlabeled (**a**–**c**) and ^13^C^15^N labeled (**d**, **e**) His (**a**, **d**), Gly (**b**, **e**), and Ala (**c**, **f**) by logarithmic dilution and MOPS concentration of unbuffered and MOPS-buffered CGXII media (*n*_analytical_ = 3, *n*_technical_ = 3)

In general, such ion suppression effects can be minimized by sample clean up or dilution [65–67]. Having in mind that the culture supernatant samples need to be diluted in order to fit in the linear range of the MS measurement, the dilution approach seems to be promising. In order to characterize the MOPS buffer inferences, different CGXII media, varying in MOPS concentration (0–200 mM) were prepared at logarithmic dilution levels. Media were diluted with 50% MeOH (v/v) and spiked with 6 μM amino acid standards prior to 1:2 IDMS dilution (*n*_analytical_ = 3, *n*_technical_ = 3). Figure 3 shows the relative area of the unlabeled and labeled analytes His, Gly, and Ala as a function of the dilution factor for the corresponding MOPS concentration.

The data gives clear evidence that MOPS is causing an additional ion suppression effect, which is exemplarily illustrated using the relative peak area of His (Fig. 3a). The data without MOPS (blue columns) show an increase of signal with increase of sample dilution. This is expected, since sample dilution increases ionization efficiency leading to an overall signal increase when the dilution factor is taken into account. However, samples with increasing MOPS concentration and low dilution factors show a strong signal decrease. The effect is stronger with higher MOPS concentration and low dilution factors. A similar pattern is observed for all compounds (Fig. 3b–f). As the trend of the His and Ala signals approaches saturation with higher dilution, there is a systematic deviation for each MOPS concentration at each dilution step for Gly. It seems that again Gly is particularly susceptible to ion suppression effects. Although the dilution series data suggests to use even higher dilution than 1:10^4^, this is limited by the sensitivity of the MS device, so that the quantification limit must be taken into account for cultivation supernatants and should not exceed the given dilution range.

Depending on the application, a column-free DS-FIA method may require an increased MS instrument cleaning frequency. The present samples or matrices in this study did not make it necessary to deviate from a monthly cleaning procedure of the ion source, curtain plate, orifice, and skimmer cone. This might be a benefit of dilution factors > 10^3^, the utilization of a high organic fraction in sample preparation, the waiver of additional modifier, low injection volumes of 5 μL, and orthogonal design of the ion source.

Overall, the MOPS buffer was identified as an interfering part of the sample matrix, which can cause substantial ion suppressing effects in DS-FIA. With the aid of a correspondingly strong dilution and IDMS, the negative effects of the MOPS buffer can be compensated and the method can be characterized and validated.

### Method validation

For validation purposes, all methods were evaluated by range, linearity, sensitivity, accuracy, precision, and robustness (*n*_technical_ = 4). Table 1 shows the comparison of the linear range for the amino acid quantification of the DS-FIA and SCX methods for the QqQ and QqToF mass analyzer. The linearity analysis (*n*_technical_ = 4) was extended beyond *r*^2^ with the response factor *m* and process standard deviation coefficient (*V*_C_) (Table 2). Table 3 shows the comparison of the MDL and PQL of the DS-FIA and SCX methods for the QqQ and QqToF mass analyzer. The method robustness, accuracy, and precision were validated with spiking experiments.

**Table 1:**
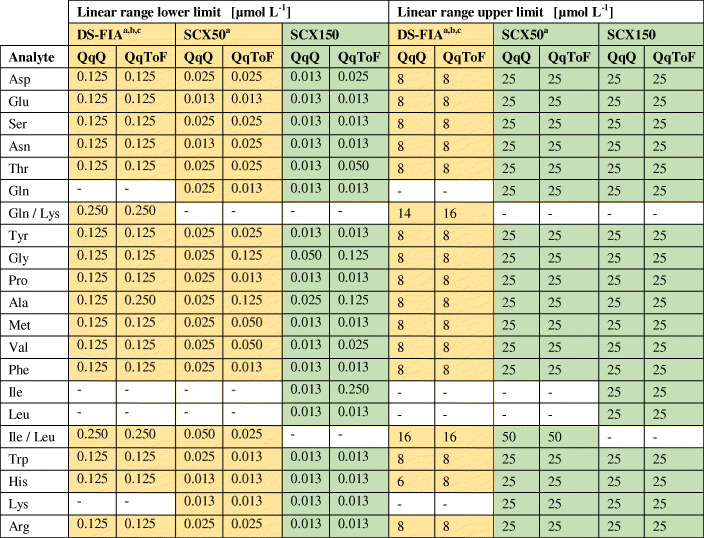
Linear range of DS-FIA, SCX50, and SCX150 methods with QqQ or QqToF mass analyzer for 19 proteinogenic amino acids. The colors indicate the observed pattern (green > yellow) with focus on the methods. Values are rounded to third decimal

^a^Summation peak of Ile and Leu due to isobaric fragmentation and no chromatographic separation

^b^Summation peak of Gln and Lys due to isobaric fragmentation and no chromatographic separation

^c^Summation peak of ^13^C^15^N Asp/Asn/Ile/Leu for single normalization of Asp/Asn and Ile/Leu, as well as ^13^C^15^N Glu and ^13^C^15^N Gln for single normalization of Glu

**Table 2:**
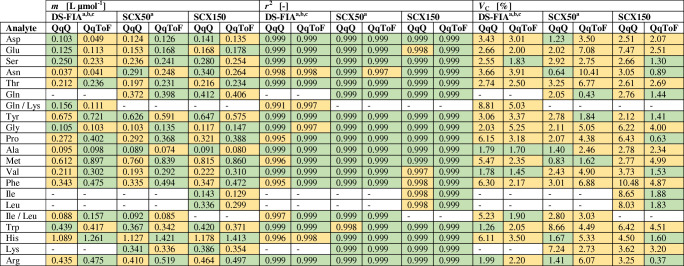
Response factor *m*, coefficient of determination *r*^2^, and process standard deviation coefficient *V*_C_ of DS-FIA, SCX50, and SCX150 methods with QqQ or QqToF mass analyzer for 19 proteinogenic amino acids (*n*_technical_ = 4). The colors indicate the observed pattern for *m* (green > yellow) with focus on the analyzer per method and the corresponding ranking for *r*^2^ (green > 0.999, 0.999 > yellow) and *V*_C_ (green < 2%, 2% < yellow). Values are rounded to third and second decimal

^a^Summation peak of Ile and Leu due to isobaric fragmentation and no chromatographic separation

^b^Summation peak of Gln and Lys due to isobaric fragmentation and no chromatographic separation

^c^Summation peak of ^13^C^15^N Asp/Asn/Ile/Leu for single normalization of Asp/Asn and Ile/Leu, as well as ^13^C^15^N Glu and ^13^C^15^N Gln for single normalization of Glu

**Table 3:**
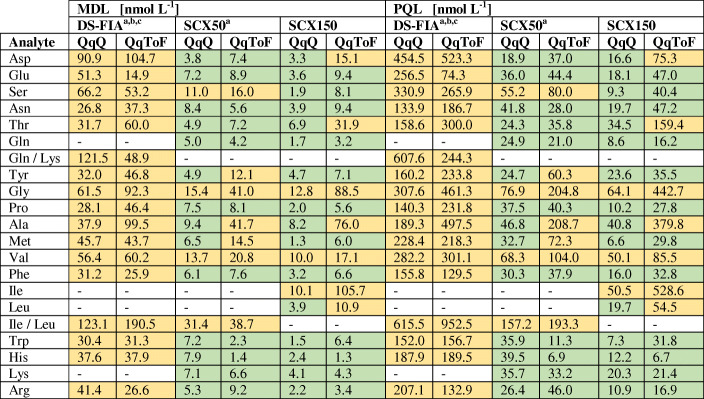
Method detection limit MDL and practical quantitation limit PQL of DS-FIA, SCX50, and SCX150 methods with QqQ or QqToF mass analyzer for 19 proteinogenic amino acids. The colors indicate the ranking for MDL (green < 10 nM, 10 nM < yellow) and PQL (green < 50 nM, 50 nM < yellow). Values are rounded to first decimal

^a^Summation peak of Ile and Leu due to isobaric fragmentation and no chromatographic separation

^b^Summation peak of Gln and Lys due to isobaric fragmentation and no chromatographic separation

^c^Summation peak of ^13^C^15^N Asp/Asn/Ile/Leu for single normalization of Asp/Asn and Ile/Leu, as well as ^13^C^15^N Glu and ^13^C^15^N Gln for single normalization of Glu

Upper and lower limits for the linear range were very similar for QqQ and QqToF with some slight better values for the QqQ only. The QqToF analyzer in general shows a higher response factor in comparison to the QqQ. Dependent on the amino acids, the QqToF might be better suited for distinguishing small changes in concentration levels. Overall, the QqQ methods showed the overall smallest MDL or PQL. Based on Eq. 2, the MDL or PQL are mainly influenced by the RSD at the lower linear range and the concentration level itself, rather than the response in IDMS. In the DS-FIA approach, i.e. without chromatography, the upper linear range boundary decreased to 8 μM for all amino acids. This could be a consequence of non-linear detector response (Fig. 1c) originating from ion suppression by co-eluting analytes. The direct comparison of *r*^2^ shows a better fit of the regression for the column methods, especially the QqToF methods. In comparison to the DS-FIA method, the column methods showed a linear range of up to 50 μM (Gln/Lys) and in general a smaller RSD for higher concentrations (see S3 in ESM_3). For single analytes, the column methods show a PQL between 7 and 443 nM and the DS-FIA methods between 74 and 523 nM.

The hypothesis for intercept of zero was rejected for 28 of 108 calibrations in 5 of 6 methods (see S4 in ESM_3), implying interferences at low concentration levels. Although an IDMS ratio *y* = 0 for *x* = 0 is expected, instrumental bias cannot be avoided. Therefore, calibration curves were not forced through zero for compensation. The selection of a non-weighted linear regression model intrinsically favors high concentrations in the modeling process. To circumvent such issues, appropriate dilution factors were chosen, i.e., analyte concentrations at the lower range could be successfully avoided.

The comparatively small response factor of Asp and Asn for the DS-FIA methods represents the above-described problem of overlapping fragmentation patterns from ^13^C^15^N Asp/Asn/Ile/Leu, resulting in a small response factor. If detector sensitivity for the determination of amino acids is of importance, the use of ^13^C labeled analytes is advised to reduce the area of labeled analyte for normalization. With regard to the ^13^C^15^N Asn/Asp challenge described above and the decreased slope for Asp and Asn, the DS-FIA-QqQ method does not lack method performance. With a smaller linear range and slope, this indicates that the number of calibration points (*k* = 9–12) and the process standard deviation show a stronger influence to the coefficient than the linear range mean and the slope.

The SCX150-QqToF method shows in general the smallest process standard deviation coefficient for all amino acids. According to Eq. 1, the comparatively large linear range, high goodness-of-fit and high response factor, results in a small error and high method performance. In general, the QqQ shows a higher sensitivity due to the MRM mode in comparison to the PI mode of the QqToF [68]. Overall, the combination of comparatively high sensitivity and analyte separation reduce the RSD at the lower linear range. A specific case represents the quantification of the two smallest amino acids Gly and Ala, which benefits from a shorter column. This might be due to excessive peak broadening by increasing column length, decreasing the signal height.

With respect to the corresponding detector, the column-free methods show up to 37 times higher MDL or PQL, while absolute single analyte PQL are still below 524 nM (Asp). Nonetheless, this is a clear performance loss for applications with low concentrated analytes and influences the choice of presented methods, if MDL or PQL is of importance. If not, the selection of appropriate dilution factors can avoid concentrations close to PQL. For example, the selection of a dilution factor between 10^3^ and 10^4^ with an IDMS factor of 2 allows to cover the concentration range of Asp between 1.05–160 mM. Depending on the buffer or analyte concentration and the resulting RSD, this range might be extended to factors between 10^2^ and 10^5^ covering 0.105–1600 mM. In addition, the short analysis time favors the rapid determination of appropriate dilution factors.

For evaluation of accuracy, precision and robustness, H_2_O, CGXII, and 200 mM buffered CGXII media were diluted 1:10^3^ with 50% MeOH (v/v) and spiked with 6 μM amino acid standards prior to 1:2 IDMS dilution (*n*_technical_ = 4).

The analysis of robustness and accuracy was evaluated by hypothesis tests of recovered metabolite pools (see S5 in ESM_3). The multi-comparison hypothesis tests evaluated the null hypothesis of same means for the corresponding amino acid concentration in two groupings: (a) to evaluate robustness, one method for different media was used and (b) one medium with different methods was used to evaluate accuracy (see S7, S8, S9, S10 in ESM_3).

For the mean concentration of the single analysis method for different media (a), the ANOVA did reject the null hypothesis for Asn with DS-FIA-QqToF method between H_2_O and 200 mM buffered CGXII medium, while the post hoc test did not. This is probably based on the use of ^13^C^15^N isotopes for IDMS and described above. Interestingly, this is not evident for Asn with DS-FIA-QqToF method for H_2_O and unbuffered CGXII as well as unbuffered CGXII with buffered CGXII. Regarding the mean concentration of all methods per matrix, the ANOVA and Holm-Bonferroni method did not reject the hypothesis of same means for all analytes and media.

**Fig. 4:**
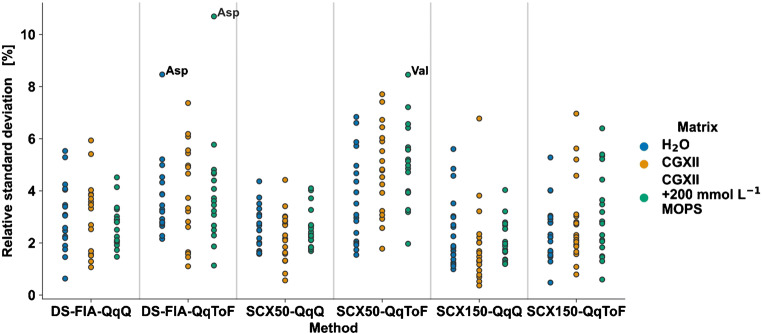
Comparison of method precision by RSD for DS-FIA, SCX50, and SCX150 methods with QqQ or QqToF mass analyzer. H2O, CGXII, and 200 mM buffered CGXII media were diluted 1:10^3^ with 50% MeOH (v/v) and spiked with 6 μM amino acid standards prior to 1:2 IDMS dilution (*n*_technical_ = 4)

The precision was evaluated by RSD (see S6 in ESM_3) with an acceptance threshold of RSD < 20%. The analysis of the RSD for all amino acids per method and media are displayed in Fig. 4. All analytes show an RSD < 12% for the spiked amino acid concentrations independent of method and media. In general, the QqQ methods showed a lower RSD (see Fig. 4) which is in consistent with recently published work evaluating QqQ and QqToF analyzer for metabolomics [68].

The validation showed that the DS-FIA method can handle cultivation supernatant samples which contained 200 mM MOPS. Due to the short analysis time and overall low RSD, the DS-FIA-QqQ method is selected for the analytical workflow.

## Case study

The newly developed and validated DS-FIA method was applied for performance characterization of a library of 96 *C. glutamicum* strain variants producing His, which originated from random mutagenesis. Thanks to the short run time of 1 min, 96 cultivation samples could be measured with 3 technical replicates in less than 5 h. The analysis with the SCX150 method would have taken up to 168 h (approx. 7 days) representing a major obstacle. The validated DS-FIA method now allows for the rapid quantification of proteinogenic amino acids, solving the analytical bottleneck in strain library characterization and bioprocess development.

**Fig. 5:**
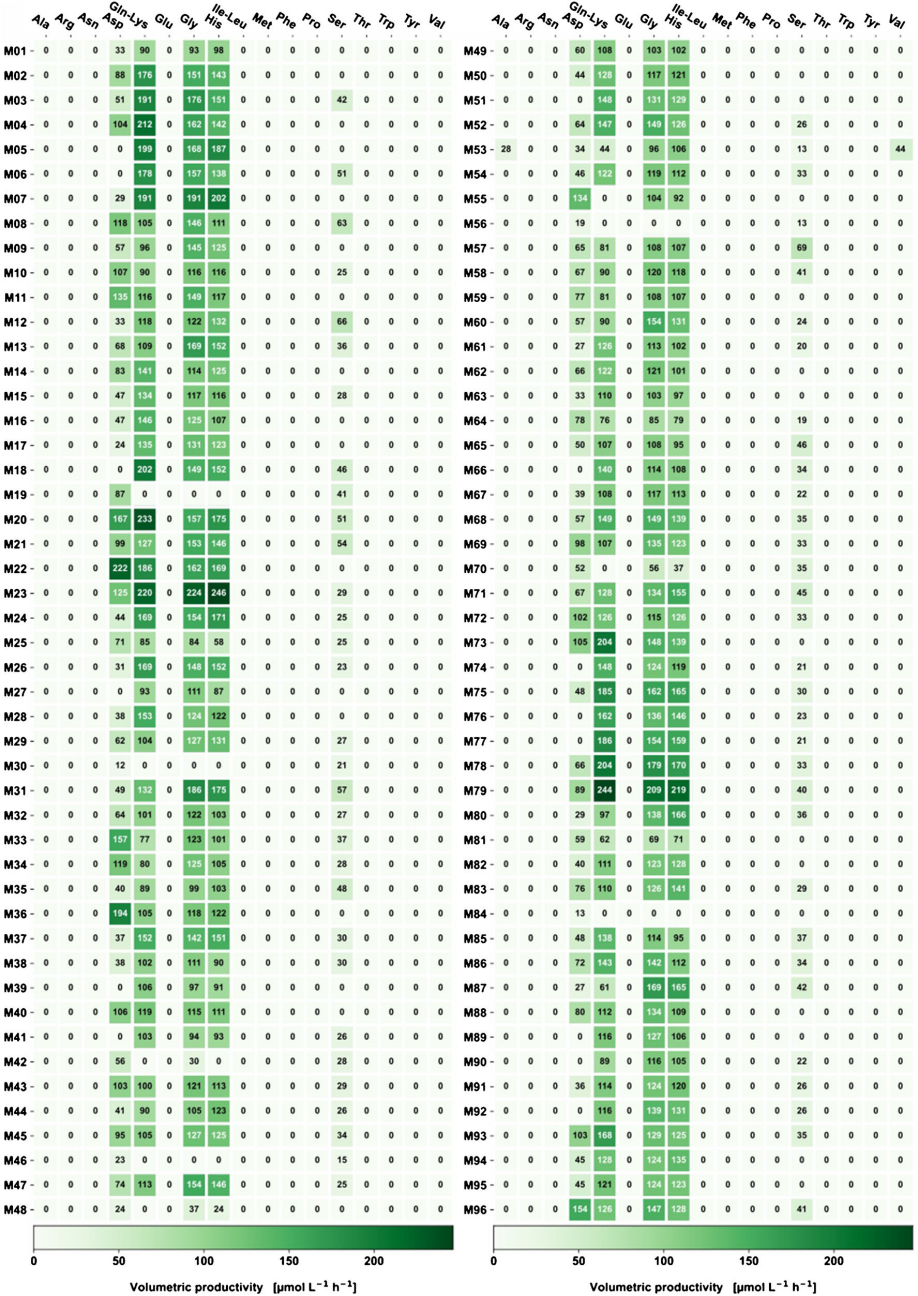
Screening of 96 His-producing *C. glutamicum* strains by DS-FIA-MS/MS. The color intensity represents the *P*_V_ of the corresponding amino acid with regard to batch cultivation time. The most promising producers M05, M07, M20, M22, M23, M24, M78, and M79 were selected by the *P*_V_ of main product His and side product Gly

In a first round of characterization, all 96 strains were cultivated in microscale cultivation, using two BioLector cultures of 48 strains each in 200 mM MOPS-buffered CGXII medium. A robotic system was used to sample the cultivations at the endpoint and the samples were centrifuged at 3320 g for 5 min at 20 °C. The obtained supernatants were diluted 1:10^3^ with 50% MeOH, subsequently diluted in a 1:2 ratio with ^13^C^15^N labeled isotope standards for IDMS and 19 amino acids were quantified with the DS-FIA method (see Fig. 5).

To evaluate the strain performance, the final value of *P*_V_ was selected. In contrast to the titer only, the *P*_V_ also includes information about potential lag-phase and total cultivation time.

In general, the His-producing *C. glutamicum* strain library shows a broad spectrum of high and low producer strains. Besides the main product His, several other amino acids like Asp, Gln/Lys, Gly, and Ser were found as by-products. Their presence could be used for metabolic engineering in order to channel the carbon flus towards the main product. Strikingly, all producers of relevant amounts of His showed side product formation of Gly in equimolar amount. The biosynthetic pathway to His might explain this, where 5-aminoimidazole-4-carboxamide ribonucleotide (AICAR) is an important intermediate. In order to further convert it along the His biosynthesis, tetrahydrofolate (THF) is formed by the AICAR formyltransferase (PurH) in the purine biosynthesis. In order to regenerate the THF acceptor the C1-metabolism is required, finally generating one Gly equivalent per His produced. Since *C. glutamicum* is not able to activate Gly as a carbon substrate, it starts to accumulate in stoichiometric amounts. Hence, management of THF redox state and Gly metabolism is identified as a primary optimization target for metabolic engineering in *C. glutamicum* to enhance His formation.

From results of the His strain library (Fig. 5), the His *P*_V_ was determined in the range of 0–246 μM h^−1^ (see S1 in ESM_4). The eight best performing mutants were: M23, M79, M07, M05, M20, M24, M78, and M22 covering a performance range of His *P*_V_ from 169 ± 1 up to 246 ± 6 μM h^−1^. For all top producer strains, Gly is produced in stoichiometric amounts representing a primary optimization target.

Besides its ability to obtain quantitative strain information, the major advantage of the DS-FIA-MS/MS method is the short analysis time. The analysis of 96 supernatant endpoint samples in three technical replicates would have taken 7 days with the SCX150 method and 2.8 days with the SCX50 method. The DS-FIA methods allows for an analysis time of 4.8 h.

**Fig. 6:**
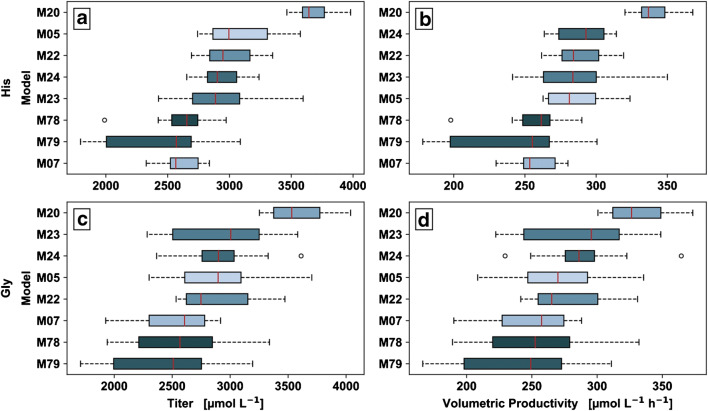
Titer (**a**, **c**) and volumetric productivity (**b**, **d**) of His (**a**, **b**) and Gly (**c**, **d**) ranked by descending median (*n*_biological_ = 4, *n*_technical_ = 3) for the most promising producer M05, M07, M20, M22, M23, M24, M78, and M79. The boxplots show the minimal and maximal values, outliers, and the 25%, 50%, and 75% quartiles for the corresponding distribution. Mutants are identically colored throughout the rankings

The eight best producers were subsequently cultivated with integrated pre-culture under identical conditions in the microcultivation platform with a reasonable number of biological and technical replicates to provide statistical evidence. The cultivation data of the replicate cultivation is shown in Chart 1 in ESM_5. The ranking of the eight producers based on His and Gly *P*_V_ is shown in Fig. 6. The box plots show the minimum and maximum values without outliers as whiskers, as well as the 25%, 50%, and 75% quantiles of the distribution (*n*_biological_ = 4, *n*_technical_ = 3). Mutants are identical colored throughout the rankings.

The box plots of the *P*_V_ and titer show the descending ranking by median. The His ranking of M20, M24, M22, M23, M05, M07, M78, and M79 by *P*_V_ mean ranges from 243 ± 43 up to 340 ± 14 μM h^−1^ (see S2 in ESM_4). The Gly ranking of M20, M24, M23, M22, M05, M78, M07, and M79 by *P*_V_ mean ranges from 238 ± 47 up to 330 ± 23 μM h^−1^ (see S2 in ESM_4). ANOVA and post hoc tests did reject the null hypothesis of same means of the His and Gly titer and His *P*_V_ for M20 to the rest of the producer (see S3, S4 in ESM_4).

In general, the specific technical error is smaller compared to the biological error and high-performing strains showed a smaller biological and technical error (see Chart 2 in ESM_5). In addition, Gly showed a lower detector response, resulting in a higher error.

The results show that the strain ranking was not dependent on the selected performance parameter, i.e., *P*_V_ or titer, respectively. Nevertheless, the equimolar production of Gly can be confirmed. Thus, a strong His producer of this strain collection is also a strong Gly producer, independent of the performance parameter selected. Overall, the producer M20 shows the highest *P*_V_ and titer for His and Gly and their performance are clearly separated from the other mutants.

In conclusion, the producer M20 represents the most promising producer of the full library and should be considered for a more comprehensive bioprocess development study in laboratory-scale batch and subsequently fed-batch experiments. Although the total number of bioreactor runs at laboratory scale will decrease, the number of process samples per cultivation will increase at the same time, so that the application of the DS-FIA method would still be beneficial for an increased sampling frequency at laboratory scale. In addition, due to the ongoing development in automated sample processing devices for microscale as well as laboratory-scale bioreactors, the sampling frequency, and thus, the total number of process samples is expected to increase. This demands for analytical procedures providing sufficient throughput, replicates, and quantitative information. At this point, the use of the DS-FIA method will become highly beneficial.

## Conclusion

The validated methods in this study present viable options for the determination of amino acids in cultivation supernatants with high throughput and quantitative nature of the data. The column-based methods SCX50 and SCX150 are especially suited for applications with the need for chromatographic resolution and low quantitation limit, but are limited in the throughput.

The DS-FIA methods are a powerful alternative to classical column-based rapid chromatography applications without a compromise in terms of quantitative quality of the data. Due to a large sample dilution of the supernatant samples and the use of labeled isotope standards, matrix or ion suppression effects can be minimized or even excluded. Quantification options are available with almost the same precision and accuracy. The throughput is accelerated by a factor 14–35 compared to the SCX methods presented in this study and a factor of 75 compared to the previously published method. The method can be used to address questions of metabolic engineering and bioprocess development. Future work will focus to extend the metabolite spectrum beyond amino acids and derivatives thereof, in order to broaden the scope of the method to cover a large set of metabolic intermediates.

## Supplementary information

ESM 1 (XLSX 15 kb)

ESM 2 (PDF 732 kb)

ESM 3 (XLSX 676 kb)

ESM 4 (XLSX 99 kb)

ESM 5 (PDF 286 kb)

## Data Availability

Additional supporting information may be found in the online version of this article at the publisher’s website.
