# Supplementary material for: Need for speed: evaluation of dilute and shoot-mass spectrometry for accelerated metabolic phenotyping in bioprocess development
Source: Anal Bioanal Chem. 2021 Mar 31;413(12):3253–68. doi: 10.1007/s00216-021-03261-3 (PMC8079306; doi:10.1007/s00216-021-03261-3)
Supplement: Supplementary file 2 — (PDF 732 kb) [file 216_2021_3261_MOESM2_ESM.pdf]

ESM2

Product Ion Spectra - Quadrupole-Time of Flight

Analytical and Bioanalytical Chemistry

Need for speed: Evaluation of Dilute and Shoot-Mass Spectrometry for accelerated metabolic phenotyping in bioprocess development

Alexander Reiter[1,2], Laura Herbst[1,2] , Wolfgang Wiechert[1,3], Marco Oldiges[1,2]

[1] Forschungszentrum Jülich GmbH, Institute of Bio- and Geosciences, IBG-1: Biotechnology, Jülich 52425, Germany

[2] RWTH Aachen University, Institute of Biotechnology, Aachen 52062, Germany

[3] RWTH Aachen University, Computational Systems Biotechnology, Aachen 52062, Germany

Corresponding author: Prof. Dr. Marco Oldiges, mail: m.oldiges@fz-juelich.de, phone: +49 2461 61-3951, fax: +49 2461 61-3870

The following spectra show the relative signal intensity over m/z. The collision energy ramp ranged from +5 to +100V. The m/z were averaged by first decimal for plotting purposes. Relative signal intensities below 1% were excluded.

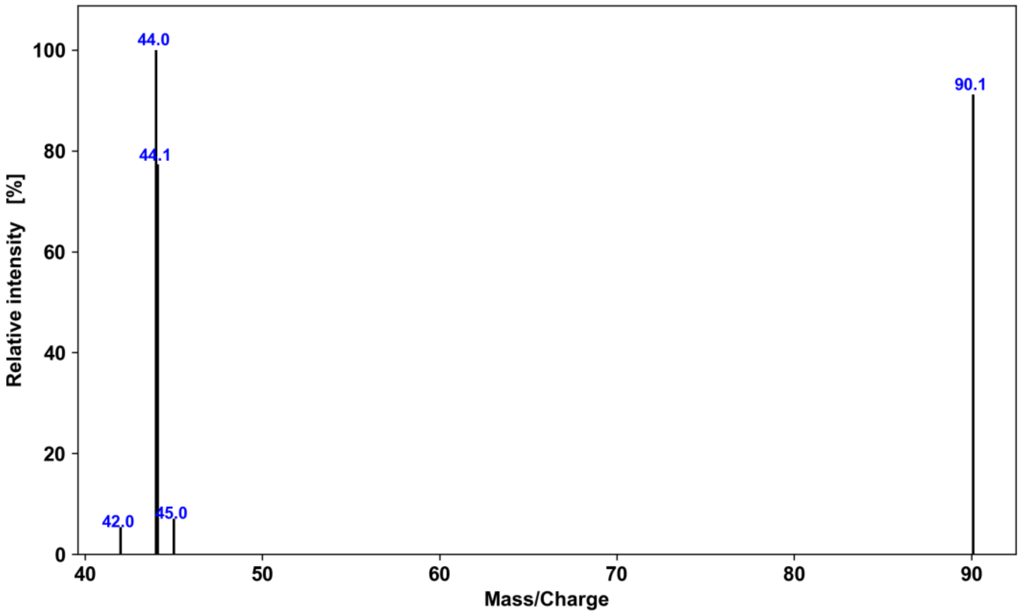

Chart 1 The product ion spectrum of the $[M+H]^+$ ion of L-Alanine

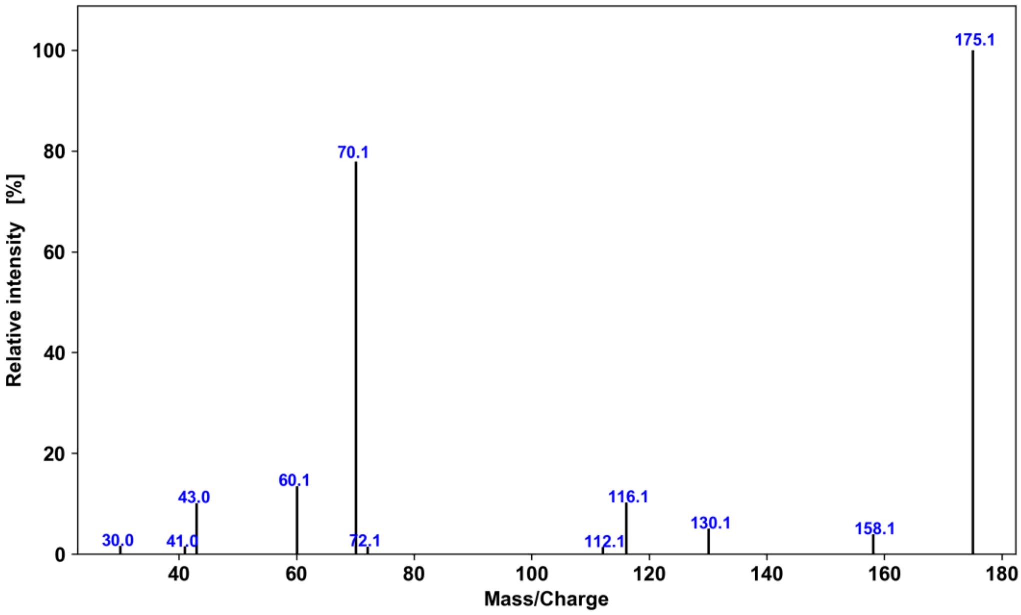

Chart 2 The product ion spectrum of the $[M+H]^+$ ion of L-Arginine

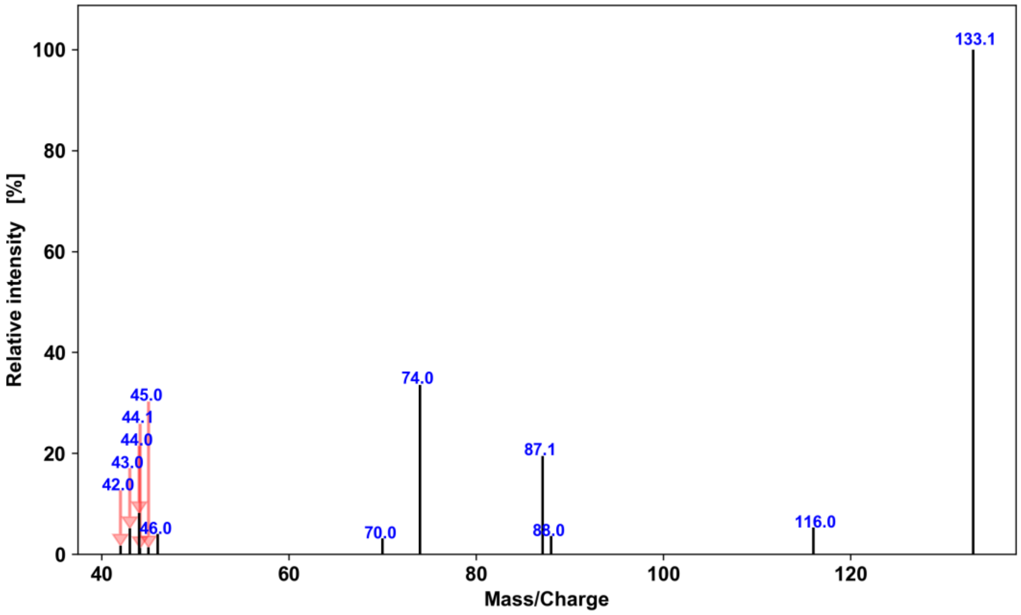

Chart 3 The product ion spectrum of the $[M+H]^+$ ion of L-Asparagine

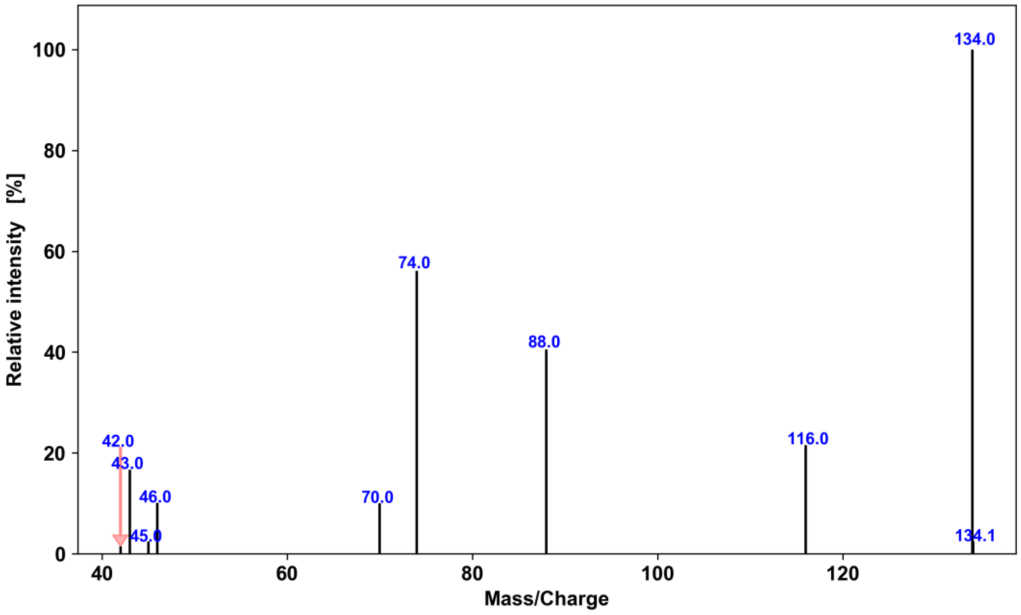

Chart 4 The product ion spectrum of the $[M+H]^+$ ion of L-Aspartic acid

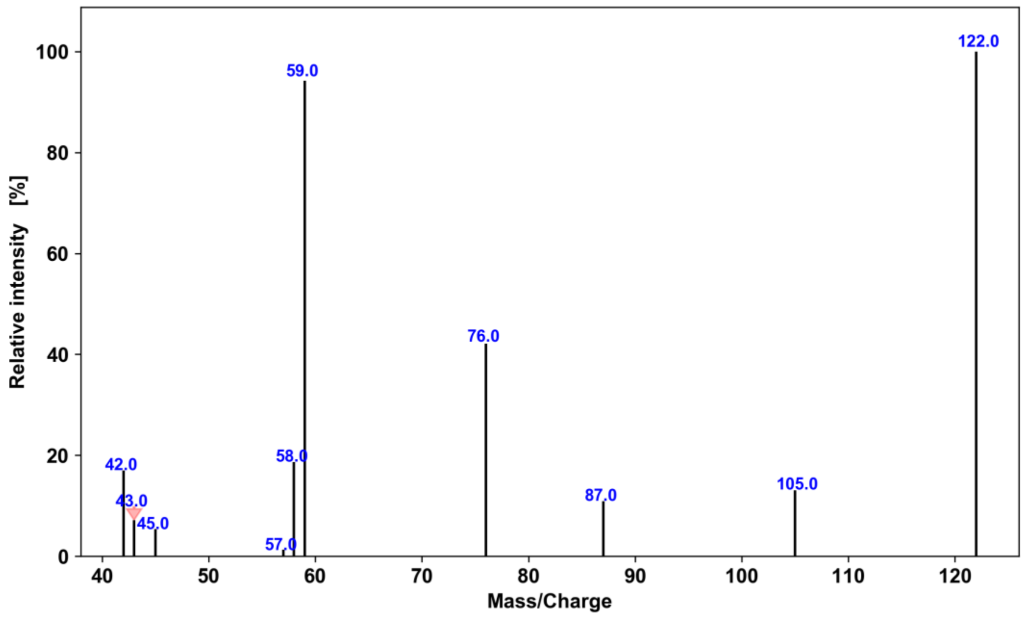

Chart 5 The product ion spectrum of the $[M+H]^+$ ion of L-Cysteine

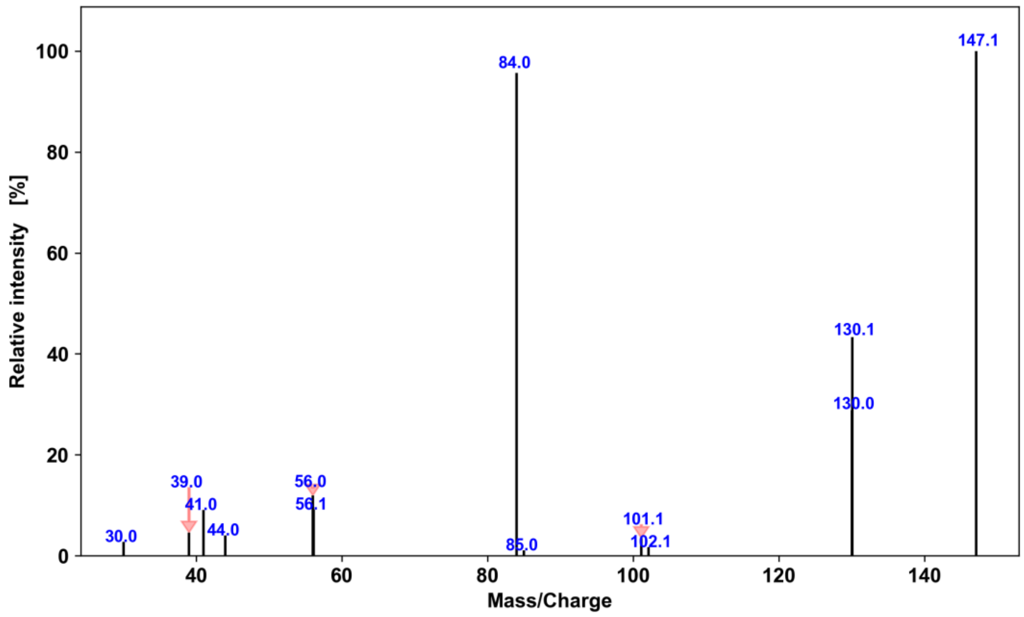

Chart 6 The product ion spectrum of the $[M+H]^+$ ion of L-Glutamine

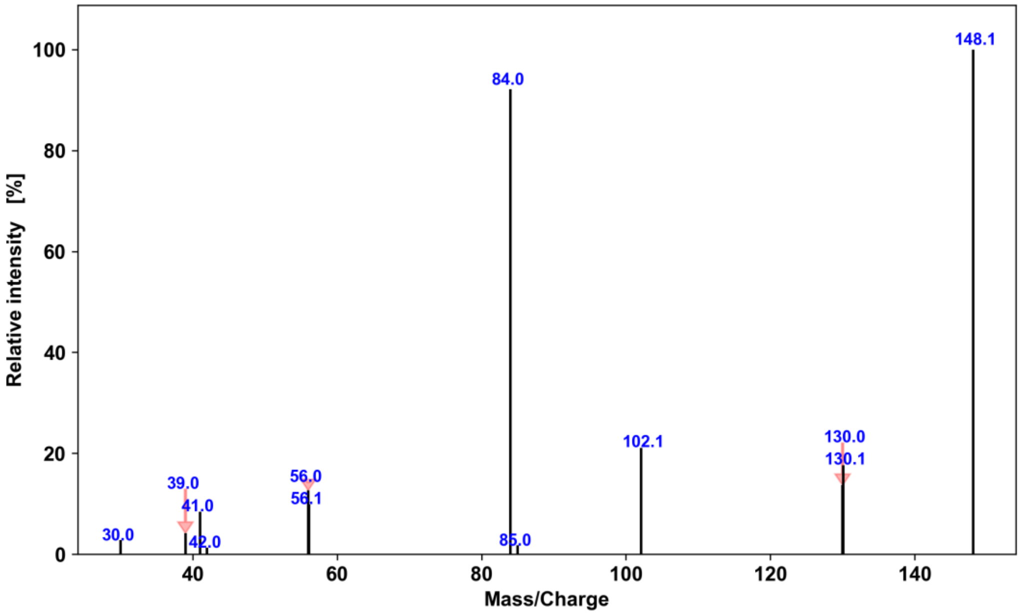

Chart 7 The product ion spectrum of the $[M+H]^+$ ion of L-Glutamic acid

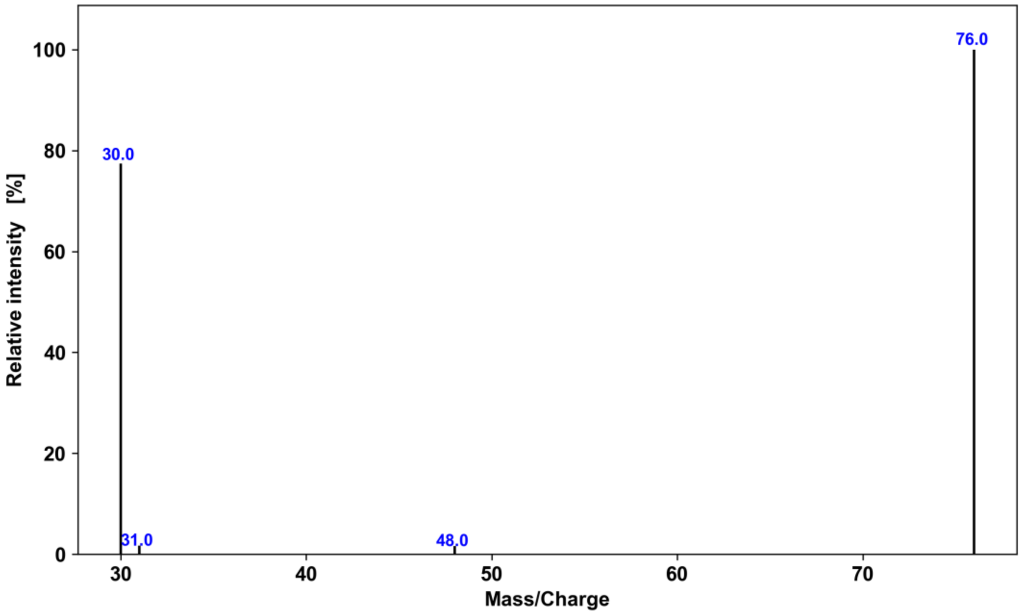

Chart 8 The product ion spectrum of the $[M+H]^+$ ion of L-Glycine

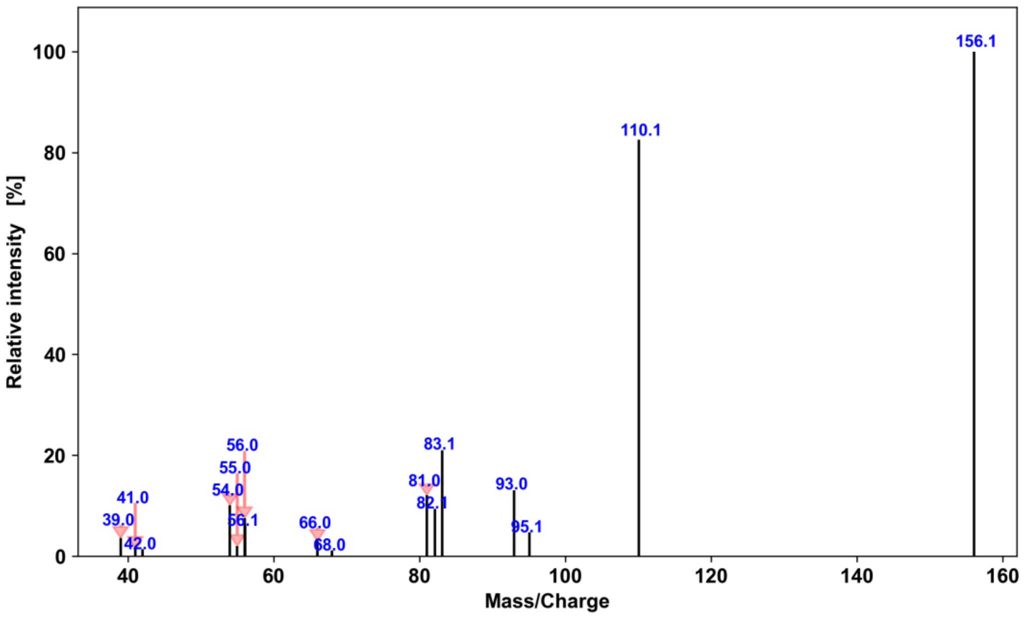

Chart 9 The product ion spectrum of the $[M+H]^{+}$ ion of L-Histidine

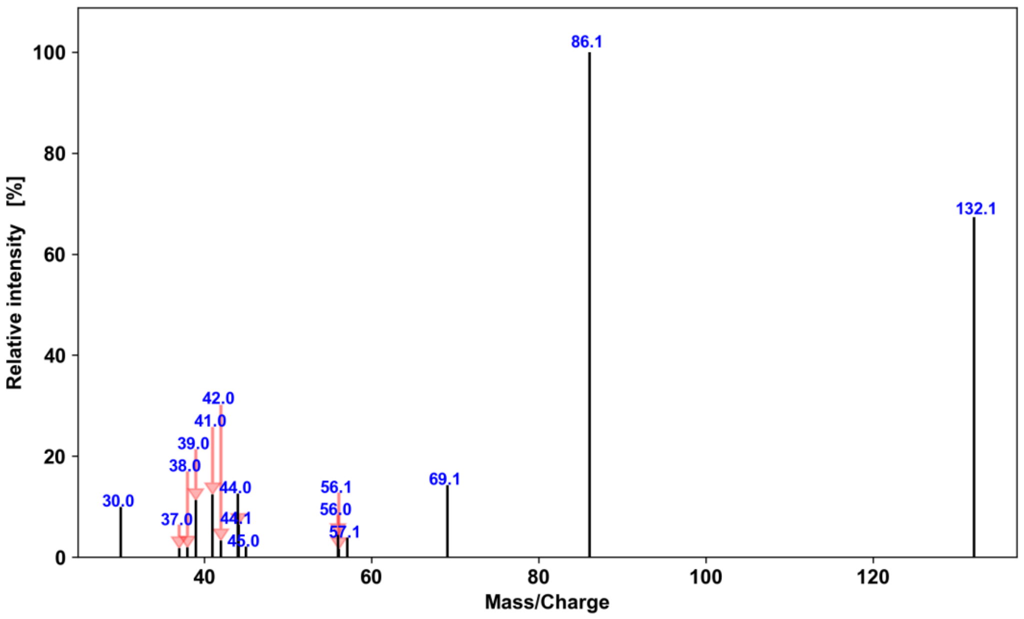

Chart 10 The product ion spectrum of the $[M+H]^{+}$ ion of L-iso-Leucine

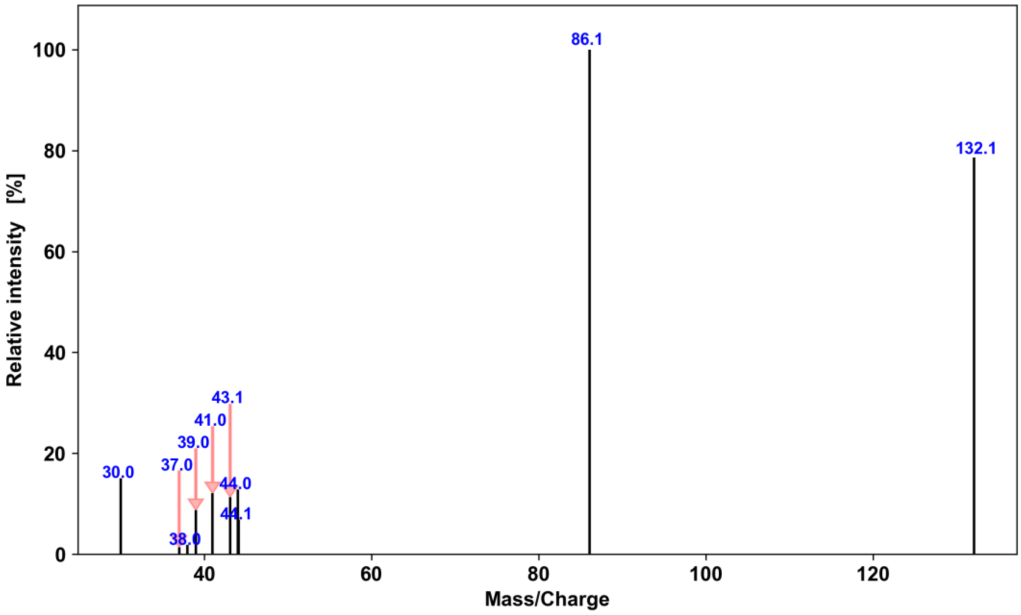

Chart 11 The product ion spectrum of the $[M+H]^+$ ion of L-Leucine

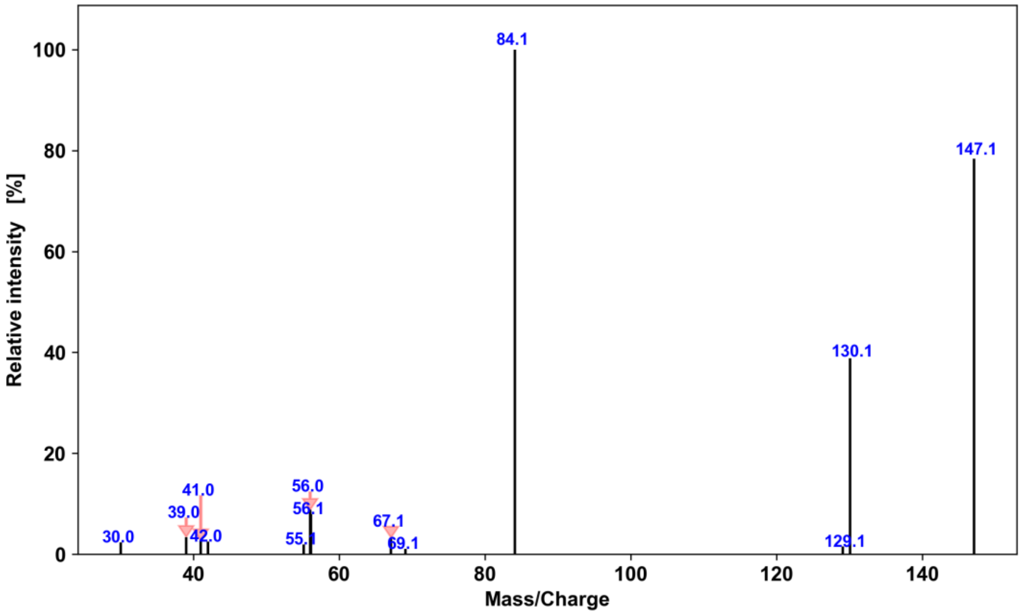

Chart 12 The product ion spectrum of the $[M+H]^+$ ion of L-Lysine

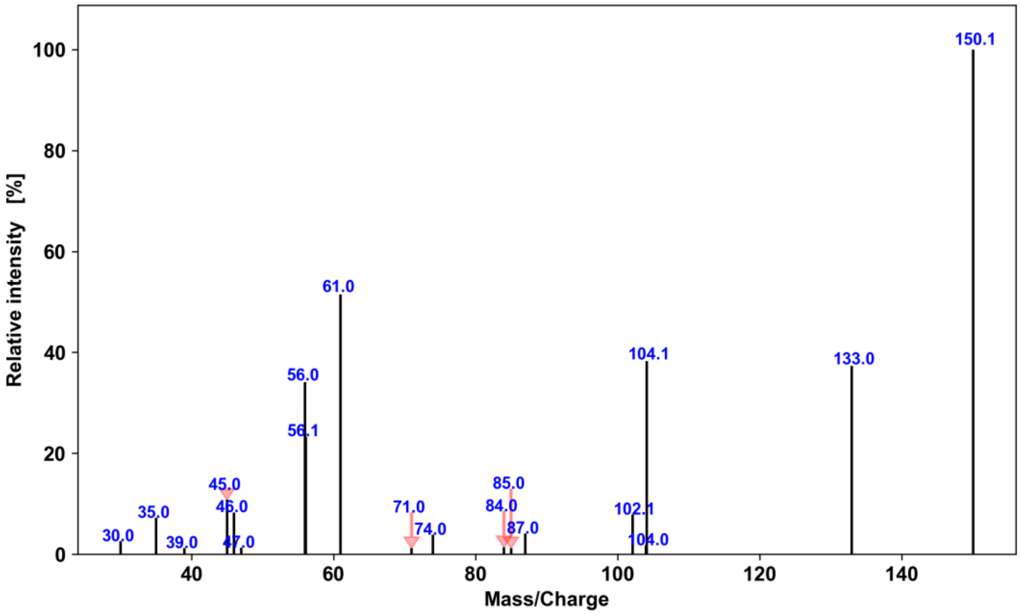

Chart 13 The product ion spectrum of the $[M+H]^+$ ion of L-Methionine

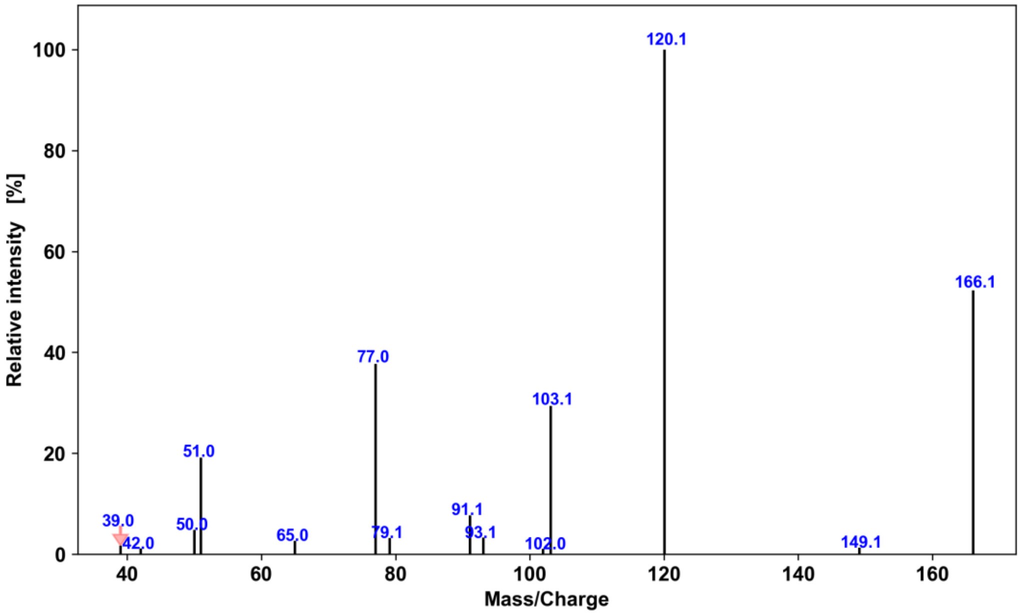

Chart 14 The product ion spectrum of the $[M+H]^+$ ion of L-Phenylalanine

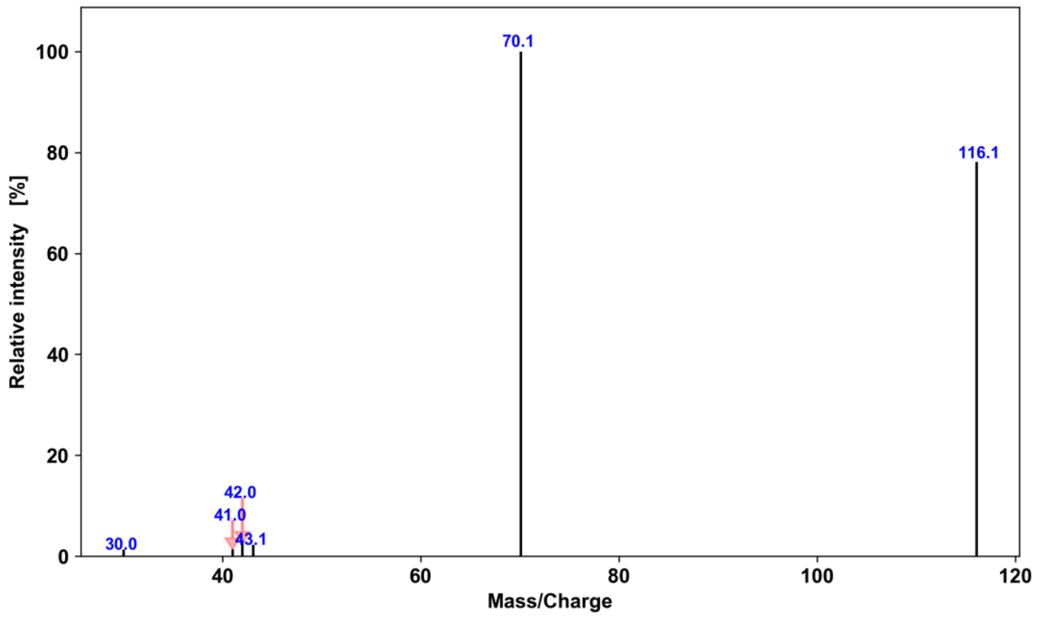

Chart 15 The product ion spectrum of the $[M+H]^+$ ion of L-Proline

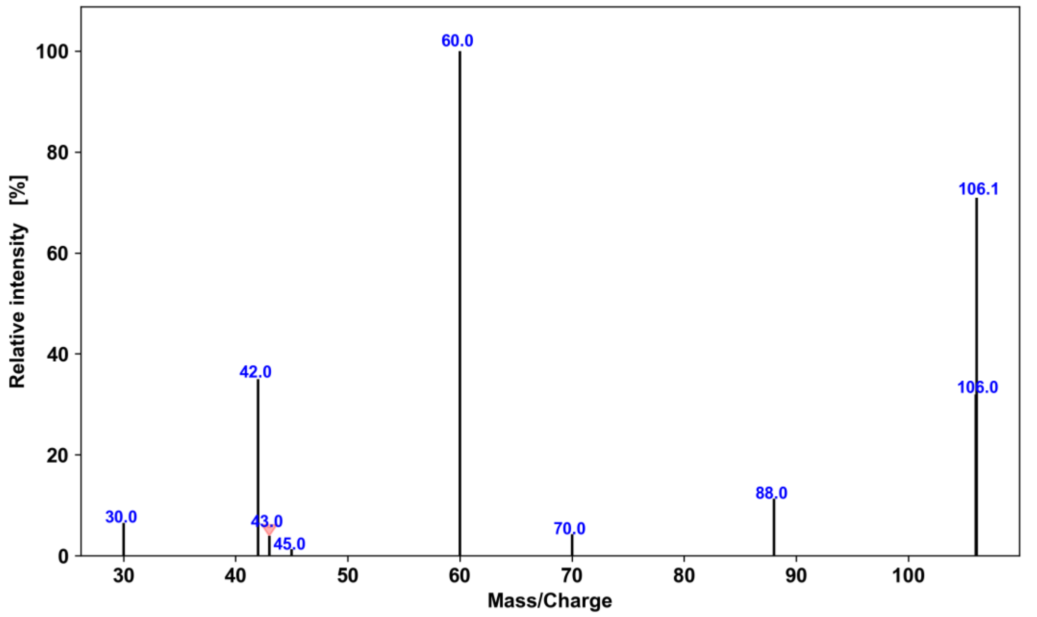

Chart 16 The product ion spectrum of the $[M+H]^+$ ion of L-Serine

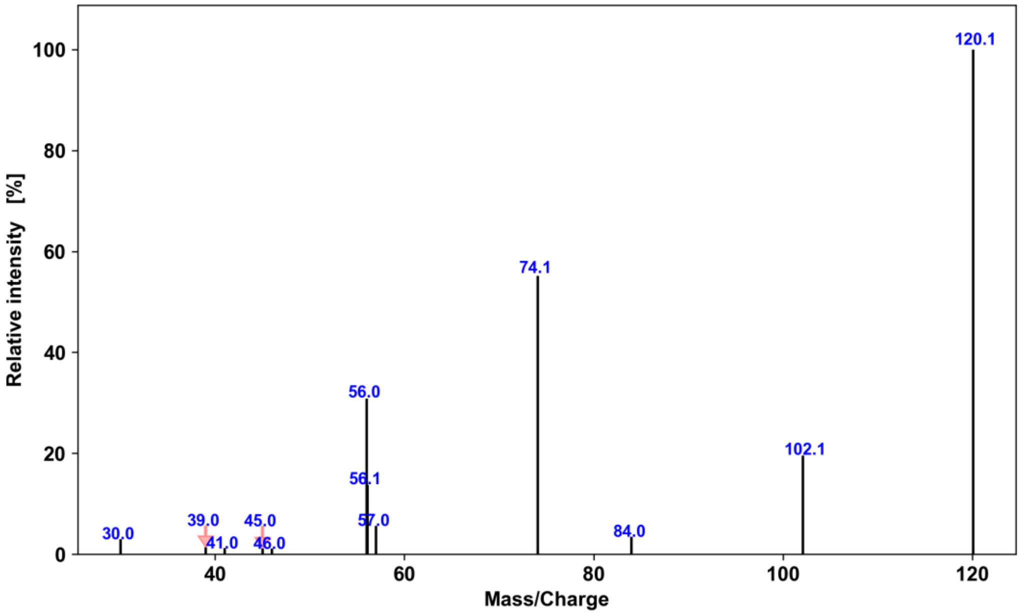

Chart 17 The product ion spectrum of the $[M+H]^+$ ion of L-Threonine

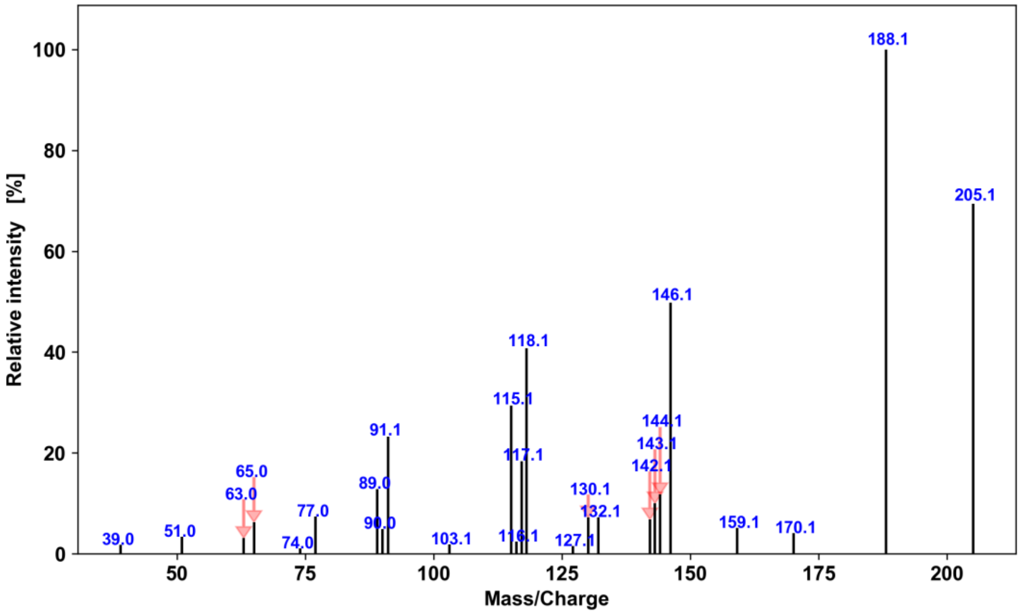

Chart 18 The product ion spectrum of the $[M+H]^+$ ion of L-Tryptophane

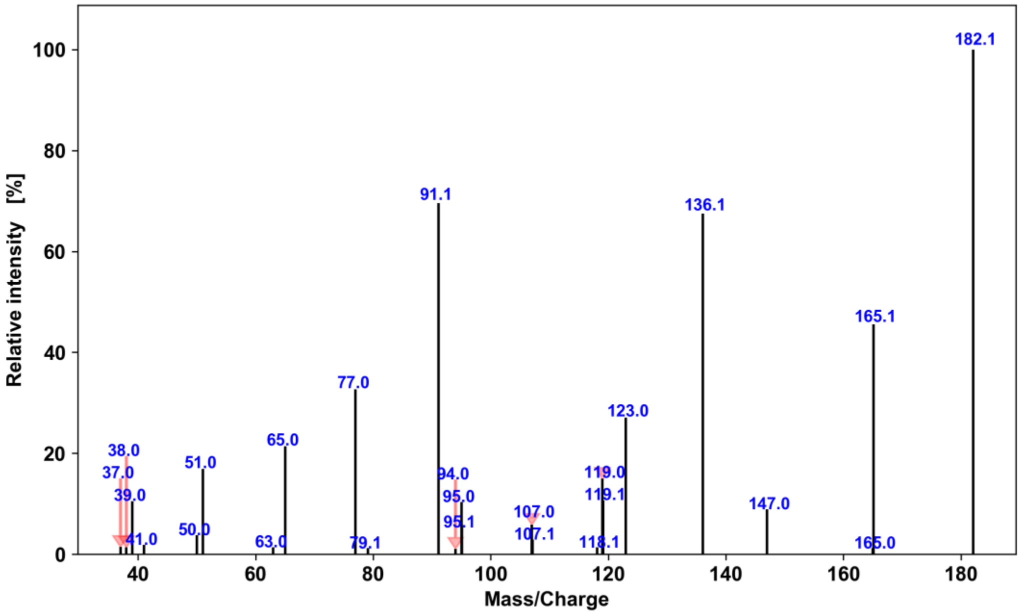

Chart 19 The product ion spectrum of the $[M+H]^+$ ion of L-Tyrosine

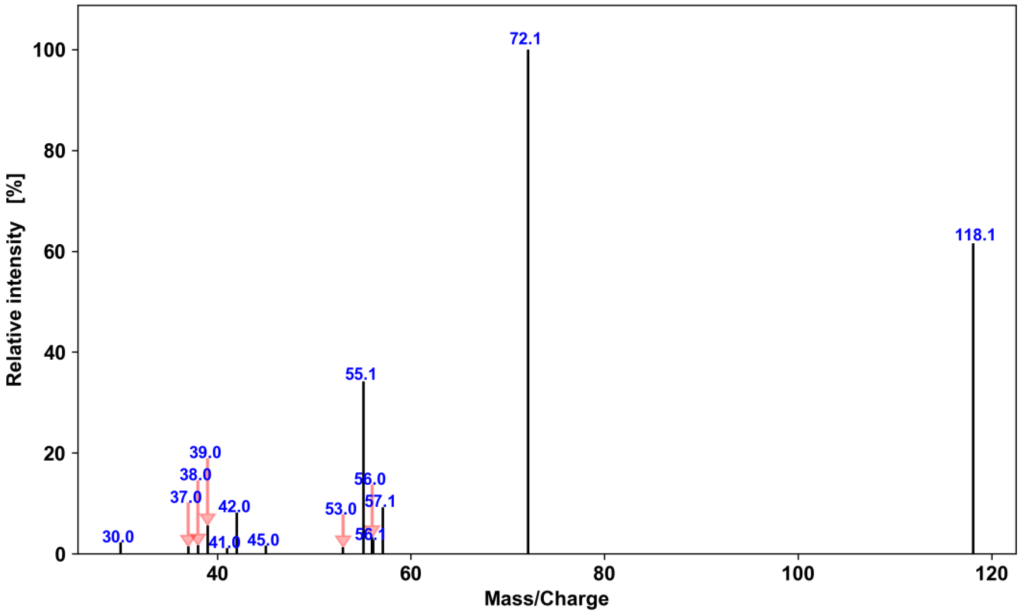

Chart 20 The product ion spectrum of the $[M+H]^+$ ion of L-Valine
