# Supplementary material for: Need for speed: evaluation of dilute and shoot-mass spectrometry for accelerated metabolic phenotyping in bioprocess development
Source: Anal Bioanal Chem. 2021 Mar 31;413(12):3253–68. doi: 10.1007/s00216-021-03261-3 (PMC8079306; doi:10.1007/s00216-021-03261-3)
Supplement: Supplementary file 5 — (PDF 286 kb) [file 216_2021_3261_MOESM5_ESM.pdf]

ESM5

Case study - Process data

Analytical and Bioanalytical Chemistry

Need for speed: Evaluation of Dilute and Shoot-Mass Spectrometry for accelerated metabolic phenotyping in bioprocess development

Alexander Reiter[1,2], Laura Herbst[1,2], Wolfgang Wiechert[1,3], Marco Oldiges[1,2]

[1] Forschungszentrum Jülich GmbH, Institute of Bio- and Geosciences, IBG-1: Biotechnology, Jülich 52425, Germany

[2] RWTH Aachen University, Institute of Biotechnology, Aachen 52062, Germany

[3] RWTH Aachen University, Computational Systems Biotechnology, Aachen 52062, Germany

Corresponding author: Prof. Dr. Marco Oldiges, mail: m.oldiges@fz-juelich.de, phone: +49 2461 61-3951, fax: +49 2461 61-3870

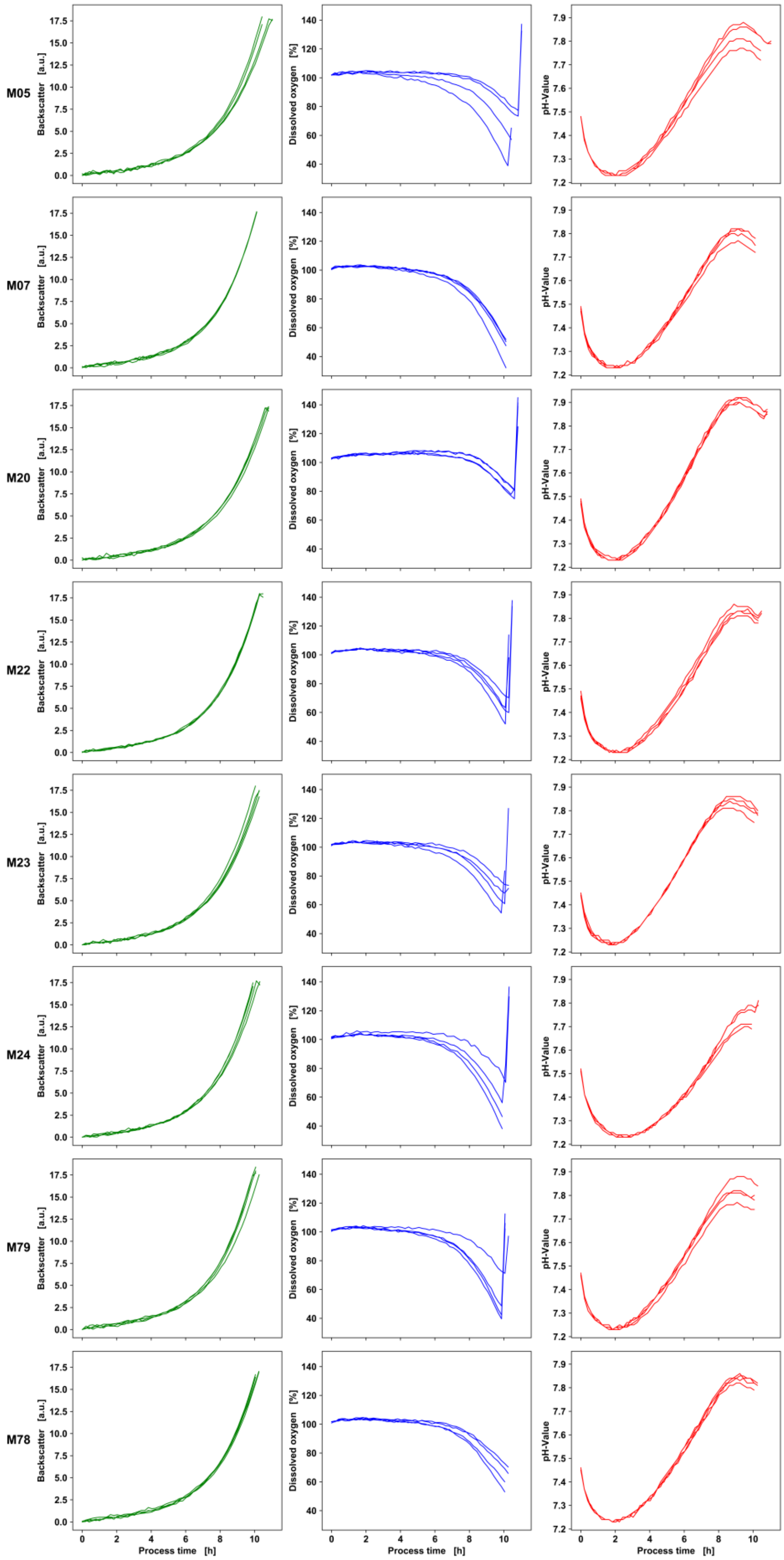

Chart 1 BioLector process data of the strains M05, M07, M20, M22, M23, M24, M78, M79 ($n_{biological}$ = 4); Backscatter, dissolved oxygen and pH-value

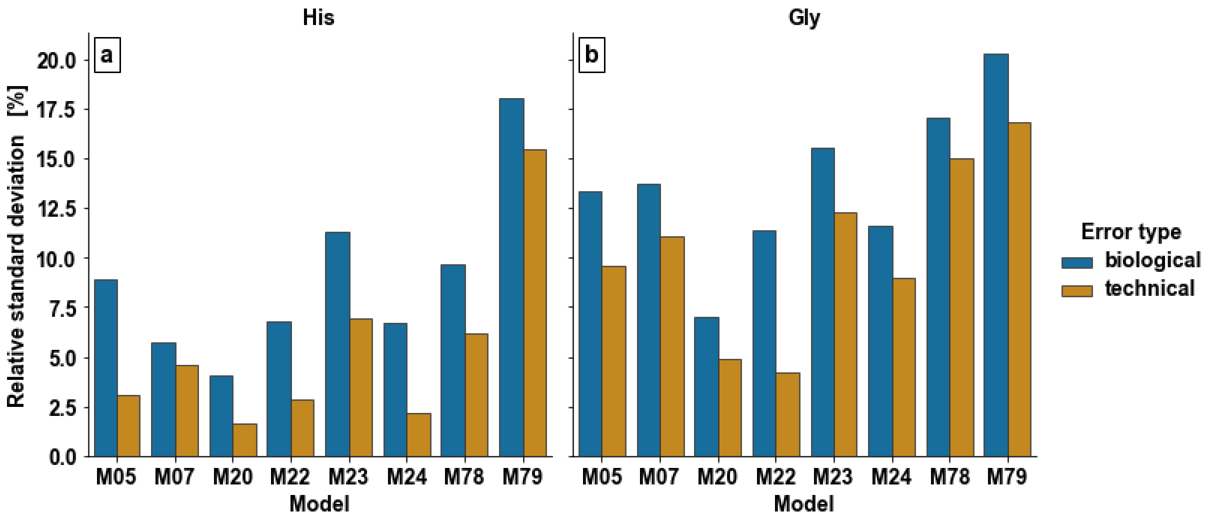

Chart 2 Error analysis of the replicate cultivation; Relative standard deviation of biological and technical replicates for the strains M05, M07, M20, M22, M23, M24, M78, M79 ($n_{biological}$ = 4, $n_{technical}$ = 4)
